# Novel high–throughput myofibroblast assays identify agonists with therapeutic potential in pulmonary fibrosis that act via EP_2_ and EP_4_ receptors

**DOI:** 10.1371/journal.pone.0207872

**Published:** 2018-11-28

**Authors:** Patrick Sieber, Anny Schäfer, Raphael Lieberherr, François Le Goff, Manuel Stritt, Richard W. D. Welford, John Gatfield, Oliver Peter, Oliver Nayler, Urs Lüthi

**Affiliations:** Idorsia Pharmaceuticals Ltd., Allschwil, Switzerland; Yokohama City University Graduate School of Medicine, JAPAN

## Abstract

Pathological features of pulmonary fibrosis include accumulation of myofibroblasts and increased extracellular matrix (ECM) deposition in lung tissue. Contractile α–smooth muscle actin (α–SMA)–expressing myofibroblasts that produce and secrete ECM are key effector cells of the disease and therefore represent a viable target for potential novel anti–fibrotic treatments. We used primary normal human lung fibroblasts (NHLF) in two novel high–throughput screening assays to discover molecules that inhibit or revert fibroblast–to–myofibroblast differentiation. A phenotypic high–content assay (HCA) quantified the degree of myofibroblast differentiation, whereas an impedance–based assay, multiplexed with MS / MS quantification of α–SMA and collagen 1 alpha 1 (COL1) protein, provided a measure of contractility and ECM formation. The synthetic prostaglandin E_1_ (PGE_1_) alprostadil, which very effectively and potently attenuated and even reversed TGF–β1–induced myofibroblast differentiation, was identified by screening a library of approved drugs. In TGF–β1–induced myofibroblasts the effect of alprostadil was attributed to activation of prostanoid receptor 2 and 4 (EP_2_ and EP_4_, respectively). However, selective activation of the EP_2_ or the EP_4_ receptor was already sufficient to prevent or reverse TGF–β1–induced NHLF myofibroblast transition. Our high–throughput assays identified chemical structures with potent anti–fibrotic properties acting through potentially novel mechanisms.

## Introduction

Idiopathic pulmonary fibrosis (IPF) mainly affects older individuals and represents a specific and severe form of chronic progressive interstitial lung disease (ILD). IPF is the most common form of ILD [1] and its cause is unknown. The median life expectancy from the time of diagnosis is approximately 3 years. In the US alone, 40,000 patients die of IPF every year [2, 3]. In recent years, several treatments have been evaluated in randomized controlled clinical trials in IPF and two anti–fibrotic agents, pirfenidone / Esbriet and nintedanib / Ofev were shown to effectively reduce the rate of lung function decline. While these approved drugs slow progression of the disease, cure is still elusive [1].

Wound repair is an essential physiological process during which damaged or dead cells are replaced and tissue architecture is restored after injury [4]. Fibrosis can develop if the wound healing process gets out of control, for instance if the normal repair process is disrupted or the tissue is continuously exposed to a damaging agent [4]. Indeed, a key driver of pulmonary fibrosis appears to be recurrent and / or persistent injury to the epithelium [3]. This can lead to increased death of alveolar epithelial cells (AEC) and / or to the emergence of a surviving AEC cell population with altered phenotype [2]. In IPF, epithelial cells are thought to secrete growth factors such as transforming growth factor–β1 (TGF–β1), which is a key mediator of fibrogenesis [5, 6] and can elicit profibrotic responses in underlying fibroblasts and related mesenchymal cells. In response to TGF–β1 stimulation fibroblasts become activated and transform into contractile α–SMA–expressing myofibroblasts that produce and secrete extracellular matrix (ECM) proteins, including collagens and fibronectin (FN). During fibrosis, myofibroblasts form aggregates that are visible in lung biopsies as fibroblastic foci and represent pathological hallmarks of interstitial pneumonias [7]. Accumulation of myofibroblasts leads to excessive deposition of ECM material, increased tissue stiffness and scarring, all of which contribute to the progressive loss of lung function. Myofibroblasts and related mesenchymal cells are considered important effector cells responsible for fibrotic destruction and distortion of lung function in IPF [2] and therefore represent promising targets for therapeutic interventions [8].

To discover new molecules targeting myofibroblast activity, we developed two novel high–throughput *in vitro* screening assays to quantify TGF–β1–induced primary normal human lung fibroblast (NHLF)–to–myofibroblast transition and ECM synthesis. The first assay, a high–content image–based assay (HCA) was used to identify cells as fibroblast or myofibroblasts by analyzing >400 features. The second assay, a multiplexed impedance and protein quantification assay, was used to identify molecules based on their ability to suppress cellular impedance changes and to attenuate α–SMA and collagen protein synthesis. Our results show that our high–throughput–compatible assays can identify novel chemical structures with anti–fibrotic properties and we demonstrate that agonists that act either via the EP_2_, the EP_4_ receptor, or as mixed EP_2_ / EP_4_ receptor agonists have the potential to attenuate myofibroblast formation, to reduce myofibroblast activity and to potentially revert a myofibroblast phenotype.

## Materials and methods

### Chemicals and reagents

For the HCA, the following reagents were used: fatty acid–free bovine serum albumin fraction V (faf BSA, Calbiochem), DAPI (Molecular Probes), Tween–20 (Sigma–Aldrich), methanol puriss. (Sigma–Aldrich), 384–well polypropylene microtiter plate (Greiner), 384–well Cell Carrier Ultra microtiter plate (Perkin Elmer), LLC backing tape (Perkin Elmer), TGF–β1 (R&D), Laminin (Invitrogen), goat serum (Invitrogen), phosphate buffered saline (PBS, containing Mg^2+^ / Ca^2+^, Gibco), penicillin–streptomycin (Invitrogen) and HCS CellMask Orange stain (Invitrogen). For the compound screening library of 1585 approved drugs, the Prestwick Chemical Library (PCL) collection of 1280 approved drugs was alimented with 305 substances from the Selleckchem FDA library (Selleckchem).

The following reagents were used for the impedance and for the RapidFire MS / MS experiments: E–plate 96 (Roche Applied Sciences), 96–well flat bottom culture plate (Corning), 96–deep well plate and 96–well skirted polypropylene polymerase chain reaction (PCR)–plate (Greiner), Oasis HLB–well plate 30 μm (5 mg sorbent / well, Oasis), Dulbecco’s phosphate–buffered saline (D–PBS, lacking Ca^2+^ / Mg^2+^), 10 x solution of HEPES and trypsin / EDTA (Invitrogen), tris hydrochloride (Tris–HCl, Applichem), benzonase nuclease (Sigma Aldrich), Tris (2– carboxyethyl) phosphine hydrochloride (TCEP, Sigma Aldrich), iodoacetamide (Sigma Aldrich), LC–MS grade formic acid 50% (Sigma Aldrich), methanol and acetonitrile CHROMASOLV Plus for HPLC (Sigma Aldrich), urea BioXtra (Sigma Aldrich), as well as thiourea ACS reagent (Sigma Aldrich). Complete Mini EDTA–free protease inhibitor tablets (Roche), ammonium bicarbonate (Fluka), sequencing grade modified trypsin and trypsin resuspension buffer (Promega). Water was purified with a Milli–Q Advantage A10 (Merck Millipore).

### Cells

*Normal human lung fibroblasts* (NHLF; donor 1, female 57 years used for impedance and protein quantification assay [IPQA] and donor 2, male 66 years used for HCA; Lonza) were cultivated in fibroblast growth medium 2 (FGM–2, Lonza), supplemented with 100 units / ml of penicillin and 100 μg / ml of streptomycin, following the supplier's instructions, and passages 3–8 were used for experiments.

*Isolation of fibroblasts from the right middle lung lobe of bleomycin–instilled 20–month old Wistar rats*. All of the experimental procedures were conducted in accordance with the Swiss animal welfare ordinance and Idorsia Animal Welfare policy on the use of experimental animals. The study was approved by the Basellandschaft Cantonal Veterinary Home Office (license no. 371). On day 0, rats (n = 3) were anesthetized with isoflurane and instilled intra–tracheally with 315 mg of bleomycin sulfate (Baxter) in a volume of 210 μl followed by 210 μl of air to equally distribute the substance in the lung. On day 28, rats were euthanized using isoflurane (5%), exsanguinated and right lung middle lobes (RML) were collected in a sterile culture dish on ice in Dulbecco's Modified Eagle's Medium (DMEM) / F12 (1:1, Thermo Fisher), minced, washed 3 times with DMEM / F12 medium and digested with 0.1% collagenase (Collagenase II–S: Invitrogen) and 15 μg / ml DNase (DNase I, Sigma) in DMEM / F12 medium in a shaking water bath at 37°C for 15 min. Digests were separated by gravity and the supernatant was transferred to a new vial supplemented with 4 volumes of ice–cold starter medium consisting of DMEM / F12 (1:1), 10% (v / v) fetal bovine serum (PAA), 1% (v / v) penicillin / streptomycin 100 U / 100 μg / ml (Thermo Fisher), and insulin / transferrin / sodium selenite (Thermo Fisher). Then, the cell suspension was passed through a cell strainer by centrifugation at 1600 rpm at 4°C for 10 min. The pelleted cells were resuspended in pre–warmed (37°C) starter medium and seeded in a cell culture dish. After 60 minutes, the medium was replaced by new starter medium. After 24 hours, the medium was exchanged for DMEM / F12 to which 10% (v / v) fetal bovine serum and 1% penicillin / streptomycin was added.

### Small molecule pharmacological agonists and antagonists of prostanoid receptors

The EP_2_ receptor selective agonists, ONO–18c, ONO–18k, and evatanepag, as well as the EP_2_ and EP_4_ antagonists, PF–04418948 and MK–2894, respectively, were resynthesized by Idorsia Pharmaceuticals Ltd according to previously described procedures [9–12]. The EP_4_ receptor agonist Merck–19a [13] was obtained from a commercial source (Cayman Chemical).

### Phenotypic HCA

Each well of a 384–well clear bottom microtiter plate was coated with 0.1% laminin solution for 60 min at 37°C and rinsed once with PBS. NHLF cells were seeded at a density of 750 cells / well in a volume of 40 μl FGM–2 medium and grown for 24 h at 37°C, 5% CO_2_ and 95% relative humidity. The plate was washed 3 times with starvation medium, e.g. fibroblast basal medium (FBM, Lonza) supplemented with 0.1% faf BSA, 100 units / ml of penicillin and 100 μg / ml of streptomycin and 250 ng / ml amphotericin B, and then starved for 24 h in 30 μl volume. NHLF were differentiated into myofibroblasts by incubation with 5 ng / ml TGF–β1 (from a stock of 20 μg / ml in 4 mM HCl, 1 mg / ml BSA) for 48 h. Compounds were prepared as 5x stocks in starvation medium and added to the starved cells to a final 1x concentration concomitant with TGF–β1 addition. The final DMSO concentration in the assay was 0.6% in all wells. 48 h after TGF–β1 addition, the medium was removed and the cells were fixed in methanol for 10 min at room temperature (RT). After fixation, cells were washed 3 times with 1 x PBS and either stored at 4°C for up to 3 days or further processed. The fixed cells were blocked with 10% goat serum in PBS, 0.25% Tween–20 for 60 min at RT. The primary antibodies, i.e., mouse monoclonal anti–α–SMA (Sigma–Aldrich) and rabbit anti–FN antibody (Sigma–Aldrich), were diluted 1:400 in 10% goat serum in PBS, 0.25% Tween–20 and the fixed cells were incubated with the antibodies for 60 min at RT. After washing the cells 3 times with 1 x PBS, 0.25% Tween–20 the detection antibodies, i.e., goat anti–mouse AlexaFluor 488 (Invitrogen) and goat anti–rabbit AlexaFluor 647 (Invitrogen) were diluted 1:1000 in 1 x PBS, 0.25% Tween–20 and incubated for 1 h at RT together with 1 μg / ml DAPI to stain nuclei and HCS CellMask Orange Stain to label the entire cell. The stained cells were washed 3 times with 1 x PBS containing 0.25% Tween–20, followed by three washes in 1 x PBS, sealed with backing tape and stored at 4°C. Images were acquired on the Opera Phenix confocal high–content screening system (Perkin Elmer) using the 20–x water immersion lens.

*Image analysis*: The image data acquired with the HCS system were uploaded to ORBIT, an open source image analysis software developed at Idorsia Pharmaceuticals Ltd (http://www.orbit.bio), and analyzed using 288 CPUs in parallel on our computing grid with the ORBIT Cell–Classifier framework using an appropriate CellProfiler [14] pipeline. DAPI stained nuclei were used to identify cells. α–SMA and FN regions were then identified and mapped to the corresponding nuclei for background correction, cell segmentation and cell feature computation to identify, classify and weigh over 400 features per cell. The computed features (S1 Table) comprise, but are not limited to, granularity, intensity, texture, area, shape and solidity of a cell in both α–SMA–and FN–expressing regions. The 0% compound effect (5 ng / ml TGF–β1) and the 100% compound effect (0 ng / ml TGF–β1) controls, were used to train and validate a support vector machine (SVM), a supervised learning model with associated learning algorithm that, once successfully validated, was applied to every cell in every well of the assayed microtiter plate. The SVM allowed autonomous weighting and classification of the characteristics and thus enabled the classifier to decide which characteristics were most relevant for the differentiation of the cell types. Features with a higher weight and a lower rank had an influence proportional to their weight on the differentiation ability of the analysis pipeline. The classification of each cell was aggregated to a cell type ratio per well, represented as the computed percentage effect value per well indicative of the degree of myofibroblast differentiation. E.g. 100% equals complete myofibroblast differentiation, 0% indicates a pure fibroblast population without any signs of differentiation.

### Impedance and protein quantification assay

#### Impedance measurements

To measure dynamic cell shape and adhesion changes during fibrotic transdifferentiation, NHLF were seeded at a density of 20,000 cells per well in FGM–2 growth medium (Lonza) containing 100 units / ml of penicillin and 100 μg / ml of streptomycin into E–plates with continued impedance signal measurement (xCELLigence system, Roche Applied Science). After overnight growth, the medium was exchanged for FBM (Lonza) supplemented with 100 units / ml of penicillin and 100 μg / ml of streptomycin and 0.1% faf BSA and cells were starved for 32 h. Compound dilution series were added and myofibroblast transition was induced by adding TGF–β1 at a concentration of 5 ng / ml, followed by continued impedance sampling for up to 48 h. For data analysis, impedance raw traces were normalized to the time point of compound addition and the baseline response (DMSO, non–TGF–β1 treated cells) was subtracted. In some cases, impedance values at defined time points were used to generate concentration–response curves to determine compound potency and efficacy using GraphPad Prism software version 7 (GraphPad Software Inc).

#### Lysis of cells for RapidFire™ MS / MS analysis

Cell culture medium was removed and cells were lysed on ice by adding 5 μl / well of pre–cooled protein extraction buffer containing 10 mM Tris–HCl (pH 8.0), 6 M urea and 2 M thiourea and shaking on ice on a plate shaker at 600 rpm for 30 min.

#### Sample preparation for MS / MS

Cell lysate was transferred to a 96–well polypropylene–PCR–plate. 15 μl / well denaturation buffer (6 M urea, 50 mM ammonium bicarbonate) and 2 μl / well of 50 mM TCEP were added and samples were denatured for 1 h at 60°C using the Veriti 96–well Fast Thermal Cycler System (Applied Biosystem) with a heated lid. Plates were allowed to cool to RT. Subsequently, 2 μl of 100 mM iodoacetamide were added per well to derivatize cysteine residues. Plates were then sealed and vortexed for 2 min in the dark and incubated for 30 min at 37°C in a thermal cycler with a heated lid. 90 μl of 50 mM ammonium bicarbonate pH 7.8 were added, followed by the addition of 10 μl of trypsin at 0.02 μg / ml. Samples were trypsin–digested overnight at 37°C in a thermal cycler with heated lid. The digest was stopped by adding 20 μl of 10% formic acid to a final concentration of 1.4% (v / v). Samples were desalted under vacuum using an Oasis HLB 96–well (30 μm particle size, 5 mg sorbent / well) reverse phase cartridge according to the manufacturer’s instructions and eluted with 170 μl of 90% acetonitrile, 0.1% formic acid. Samples were dried down with a flow of nitrogen at 40°C, left to cool to RT and re–suspended in 40 μl 5% acetonitrile, 0.1% formic acid on a plate shaker set to 1400 rpm for 5 min. Plates were centrifuged at 4,000 rpm for 10 min and 2 times 12 μl / well of supernatant were transferred into separate 96–well plates (Greiner Bio–One), sealed and stored at 4°C until analyzed by MS / MS.

#### MS / MS detection

Surrogate tryptic peptides were chosen for detection of COL1 (COL1A1), α–SMA (ACTA) and tubulin (TBA1A1) and crude synthetic peptides were purchased from Thermo Fischer. After several rounds of optimization following standard workflows [15, 16], GVVGLPGQR (COL1 / COL1A1), GYSFVTTAER (α–SMA / ACTA) and DVNAAIATIK (tubulin / TBA1A1) were chosen to be appropriate for analysis on the RapidFire™ instrument due to the absence of interfering signals in a standard C–18 reverse phase gradient. Using Skyline with 70,000 human proteins from the Unitprot database, the peptides were found to belong to the following proteins; GVVGLPGQR, COL1A1 (P02452); GYSFVTTAER, ACTA (P62736), ACTC (P68032), ACTS (P68133), ACTH (P63267) and ACTA1 (A6NL76); DVNAAIATIK, TBA1A (Q71U36), TBA1C (Q9BQE3) and 5 other tubulin entries. Although the GYSFVTTAER is not specific for ACTA, several lines of evidence suggest it is an appropriate surrogate: the magnitude of changes were similar to ACTA in Western blots and TGF–β1–induced changes were devoid of tryptic peptides covering some of the contaminating proteins. The specificity of the peptides was the same in rats. LC–MS / MS analysis was performed on a RapidFire™ RF360 system (Agilent Technologies) coupled to an API 5500 (ABSciex) with positive ion electrospray ionization. Samples were aspirated from 96–well plates into a 10–μl sample loop and loaded onto a C8 solid phase extraction (SPE) cartridge, washed for 3 s at a flow rate of 1.25 ml / min of water containing 0.1% (v / v) formic acid. Peptides were eluted from the SPE cartridge at a flow rate of 1.25 ml / min with 90% (v / v) methanol in water containing 0.1% (v / v) formic acid over 4s, directly into the mass spectrometer source. The system was re–equilibrated for 1s to the initial loading conditions at a flow rate of 1.25 ml / min. The entire cycle time was 10.5 s per sample. Detection was performed using multiple reaction monitoring (MRM) mode to monitor parent⟶product ion transitions (m / z). Transitions were 441.73⟶457.3 for GVVGLPGQR. + 2y4 COL1A1, 565.86⟶577.3 for GYSFVTTAER. + 2y8 ACTA and 508.29⟶801.48 for DVNAAIATIK. + 2y8 TBA1A. The source parameters were curtain gas (CUR) nitrogen: 20 psig; collision gas (CAD): 7 psig; ion source gas 1: 50.0 psig; ion source gas 2: 50.0 psig; ion spray voltage (IS): 5500 V; turbo heater temperature (TEM): 600°C; entrance potential (EP): 10V. Peak areas for COL1A1 and ACTA were normalized by dividing by TBA1A peak area.

### Immunoblot

The cells were washed once with 1x PBS and lysed with ice cold RIPA buffer (Sigma Aldrich) to which complete mini EDTA–free protease inhibitor cocktail (Roche) had been added to a final 1x concentration. Cell lysates were separated on a NuPAGE 4–12% Bis–Tris gel (Thermo Fisher) and transferred to a nitrocellulose membrane. The membrane was blocked overnight at 4°C in 2.5 g / l TOP Block (Juro) in 150 mM NaCl, 10 mM Tris–HCl, 0.05% Tween–20. α–SMA and α / β–tubulin were detected with mouse anti–α–SMA primary antibody (Abcam, clone [1A4]) in combination with sheep anti–mouse IgG HRP–linked secondary antibody (Amersham Biosciences) and with rabbit anti–α / β–tubulin antibody followed by incubation with HRP–conjugated donkey anti–rabbit secondary antibody. Peroxidase activity associated with immunoreactive bands was detected with the Western Lightning ECL chemiluminescence reagent (PerkinElmer) by using the LAS–4,000 imaging system (Fujifilm).

### RealTime–Glo MT viability assay

The effect of compounds on cell viability was assessed by quantifying the relative metabolic activity of cells using the non–lytic luminescence–based RealTime–Glo™ MT cell viability assay (Promega) according to the manufacturer’s instructions.

### cAMP assays

Recombinant HEK–293 cells expressing the human EP_2_ or EP_4_ receptors were used to assess cAMP stimulation. Cells were cultivated in DMEM supplemented with Glutamax (Invitrogen) and with 10% heat–inactivated FBS (GE Healthcare), collected with cell dissociation buffer (Invitrogen), and seeded in a 384–well small volume plate (Greiner) in assay buffer (1× HBSS, 20 mM HEPES, 0.2% faf BSA) at a density of 4,000 cells / well and exposed to agonist serially diluted in assay buffer containing IBMX (2 mM). After 30 min at 37°C, cells were lysed, cAMP levels were determined using the homogenous time–resolved fluorescence cAMP dynamic 1 kit (Cisbio) according to the supplier’s recommendation and fluorescence was read with a microplate reader (PHERAstar, BMG Labtech). EC_50_ values were calculated using the proprietary IC_50_Studio software (Idorsia Pharmaceuticals Ltd).

### β–arrestin recruitment assays

EP_2_ and EP_4_ receptor β–arrestin (HEK–293 human PTGER2 and PTGER4 PathHunter) assays were purchased from DiscoverX. Recombinant HEK–293 PathHunter cells were detached and seeded in 384–well plates and grown overnight in OptiMEM medium (Invitrogen). Compounds were incubated for 120 min at 37°C. Chemiluminescent PathHunter detection reagent was added according to the manufacturer’s instructions followed by luminescence quantification on a microplate reader (PHERAstar, BMG Labtech). EC_50_ values were calculated using the proprietary IC_50_Studio software (Idorsia Pharmaceuticals Ltd).

### Data analysis

The concentration that causes 50% of the maximal TGF–β1 response (EC_50_) and the concentration that causes 50% inhibition (IC_50_) were calculated by using the three parameter dose–response fit Y=Bottom+(Top–Bottom)/(1+10˄(LogEC50–X)) in GraphPad Prism. Spearman correlation was performed in GraphPad Prism for the tested small molecule compounds. Two–tailed p values <0.05 for the Spearman correlations were considered significant. Parametric Bravais–Pearson linear correlations were calculated with the open source software DataWarrior [17]. Additional calculations and statistics were performed using Microsoft Excel 2010 or GraphPad Prism 6 for graphed data.

## Results

### Supervised machine learning–based quantification of high–content confocal microscopy images of fibroblast–to–myofibroblast transition

**Fig 1:**
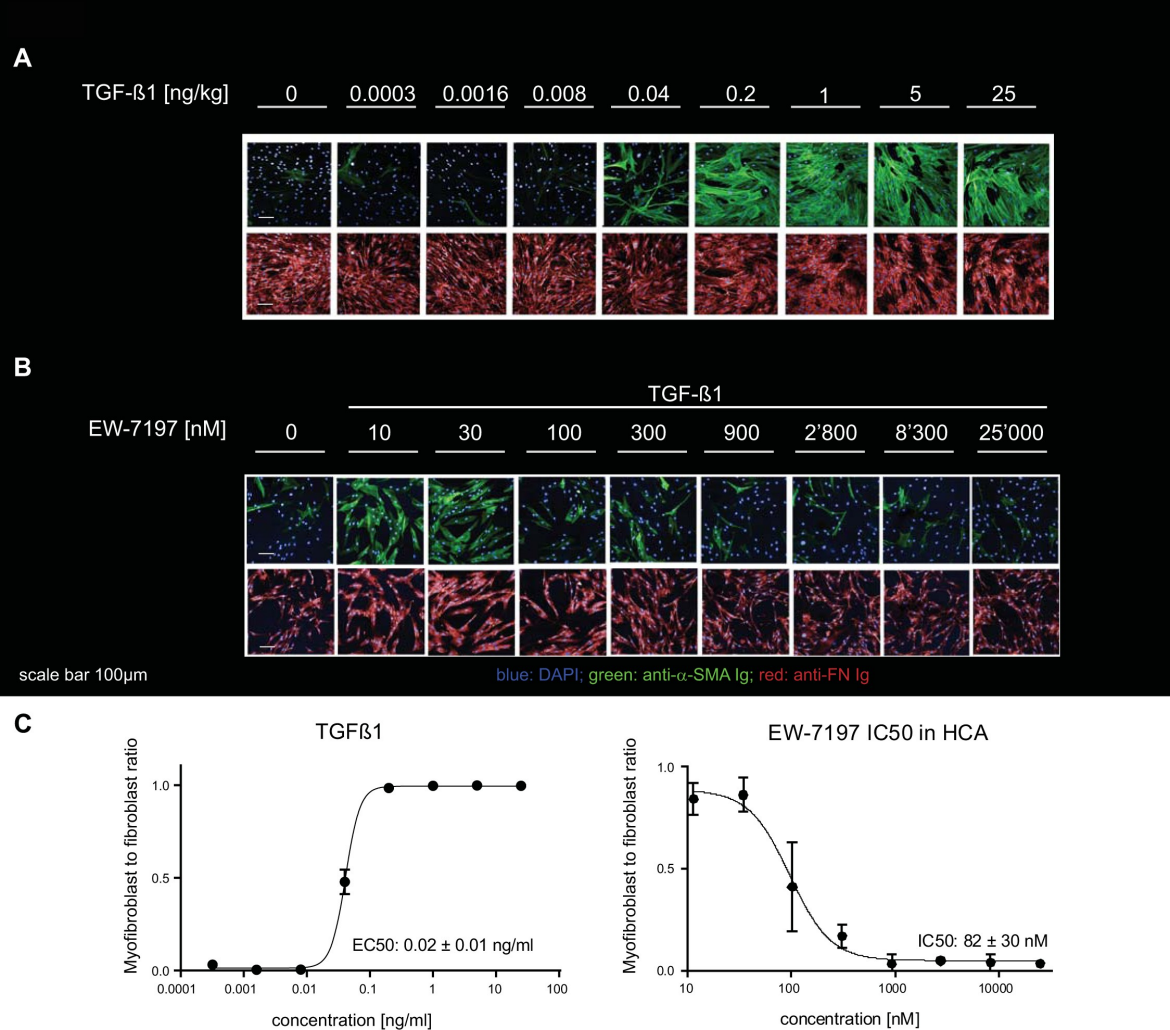
TGF–β1–stimulated normal human lung fibroblast–to–myofibroblast differentiation and inhibition thereof by using the ALK5 inhibitor EW–7197. (A) Effect of increasing concentrations of TGF–β1 ranging from 0–25 ng / ml on NHLF fibroblasts as captured by high–content confocal microscopy. (B) The effect of TGF–β1 (5 ng / ml) is inhibited by increasing concentrations of the ALK5 inhibitor EW–7197 from 10 to 25,000 nM concentration. 0 panel had no TGF–β1. (C) Concentration response curves of TGF–β1 and EW–7197 in presence of 5 ng / ml TGF–β1 were generated from the myofibroblast to fibroblast ratios as classified by the trained SVM. Curves were generated from data shown in panels A, B (n = 2). EC_50_ and IC_50_ values represent mean ± SD (n = 7). Scale bar 100 μm.

We first developed a confocal microscopy–based high–throughput compatible HCA to generate high resolution images of α–SMA, FN and DAPI positive stained myofibroblasts. These myofibroblasts were induced from primary NHLF that were grown in presence of TGF–β1 for 48 hours. The image data were analyzed using a customized CellProfiler [14] software pipeline, (see Material and Methods). A trained support vector machine (SVM) allowed autonomous weighting and classification of more than 400 computed features (S1 Table) per cell and enabled each cell to be classified as either fibroblast or myofibroblast. The training accuracy, expressed as the percentage to which a cell has been assigned to the correct class in the training set was > 97%, as determined with 10–fold cross–validation. In the HCA described here, granularity was amongst the most relevant distinguishing features to classify cells. Granularity measures the number and the distribution of objects of a certain size without segmenting them. However, all computed features were taken into account for classification. Increasing concentrations of TGF–β1 potently induced NHLF transition into myofibroblasts (Fig 1A and 1C) whereas EW–7197, a TGF–β1 receptor type I / ALK5 inhibitor [18], blocked myofibroblast formation (5 ng / ml TGF–β1, Fig 1B and 1C).

### Development of a novel multiplexed impedance and MS / MS assay

**Fig 2:**
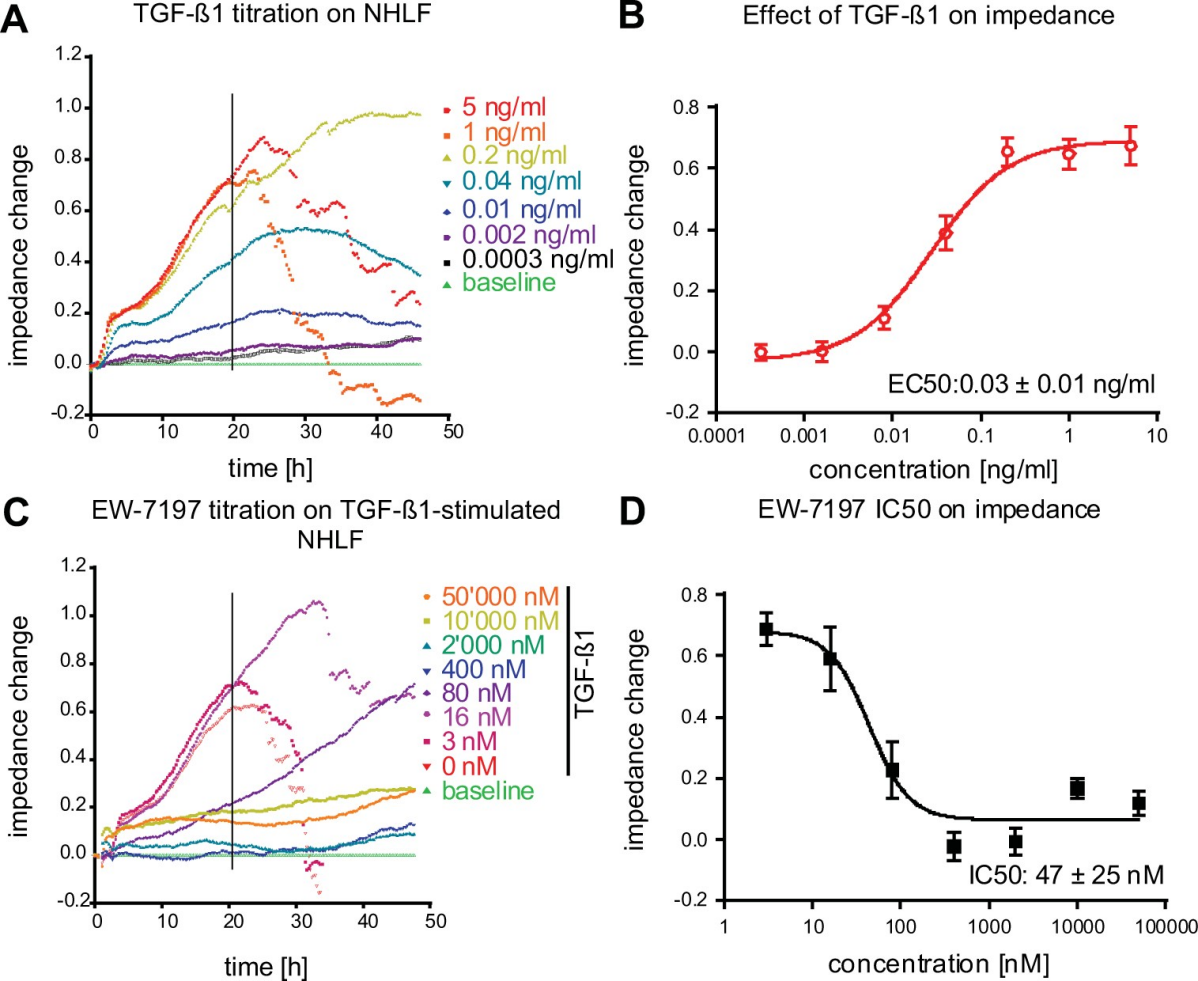
Impedance analysis of TGF–β1–stimulated fibroblast to myofibroblast differentiation and inhibition thereof with the ALK5 inhibitor EW–7197. (A) NHLF fibroblasts were stimulated at t = 0 h with different concentrations of TGF–β1 (0–5 ng / ml) and impedance responses were followed for 48 h. (C) NHLF cells were incubated with a dilution series of the ALK5–blocker EW–7197 (0–50,000 nM) and then stimulated with 5 ng / ml TGF–β1. Impedance responses were monitored for 48 h. Concentration response curves of TGF–β1 (B), and of EW–7197 in presence of 5 ng / ml TGF–β1 (D), where then generated with baseline (0 ng / ml TGF–β1) subtracted impedance values at t = 20 h post TGF–β1 addition (mean ± SD, n = 8). Representative experiments are shown in A and C.

The transition of fibroblasts into contractile myofibroblasts is also characterized by changes in cell shape and adhesion [19]. To extend and complement our HCA results, we developed a novel high throughput assay that combines quantitative cellular shape change measurements, using label–free impedance measurement [19], with MS / MS–based protein analysis (Impedance and Protein Quantification Assay: IPQA). Stimulation of NHLF fibroblasts with increasing concentrations of TGF–β1 (0–5 ng / ml; t = 0 h) lead to a transient and concentration–dependent increase in impedance (Fig 2A). For cell seeding densities ≥ 20,000 cells / well a subsequent and rapid decline of the impedance occurred typically around 24 h after TGF–β1 addition (Fig 2A). This decline reflects a contraction and partial detachment of the cell layer from the substrate, as observed by differential interference contrast (DIC) light microscopy. EC_50_ values were obtained by plotting the impedance changes 20 h after TGF–β1 stimulation. As evidenced by the changes in impedance, TGF–β1 induced a dose–dependent NHLF–to–myofibroblast formation (Fig 2). In line with the HCA findings, the ALK5 blocker EW–7197 [18] concentration–dependently inhibited the TGF–β1–induced impedance changes (Fig 2C and 2D).

**Fig 3:**
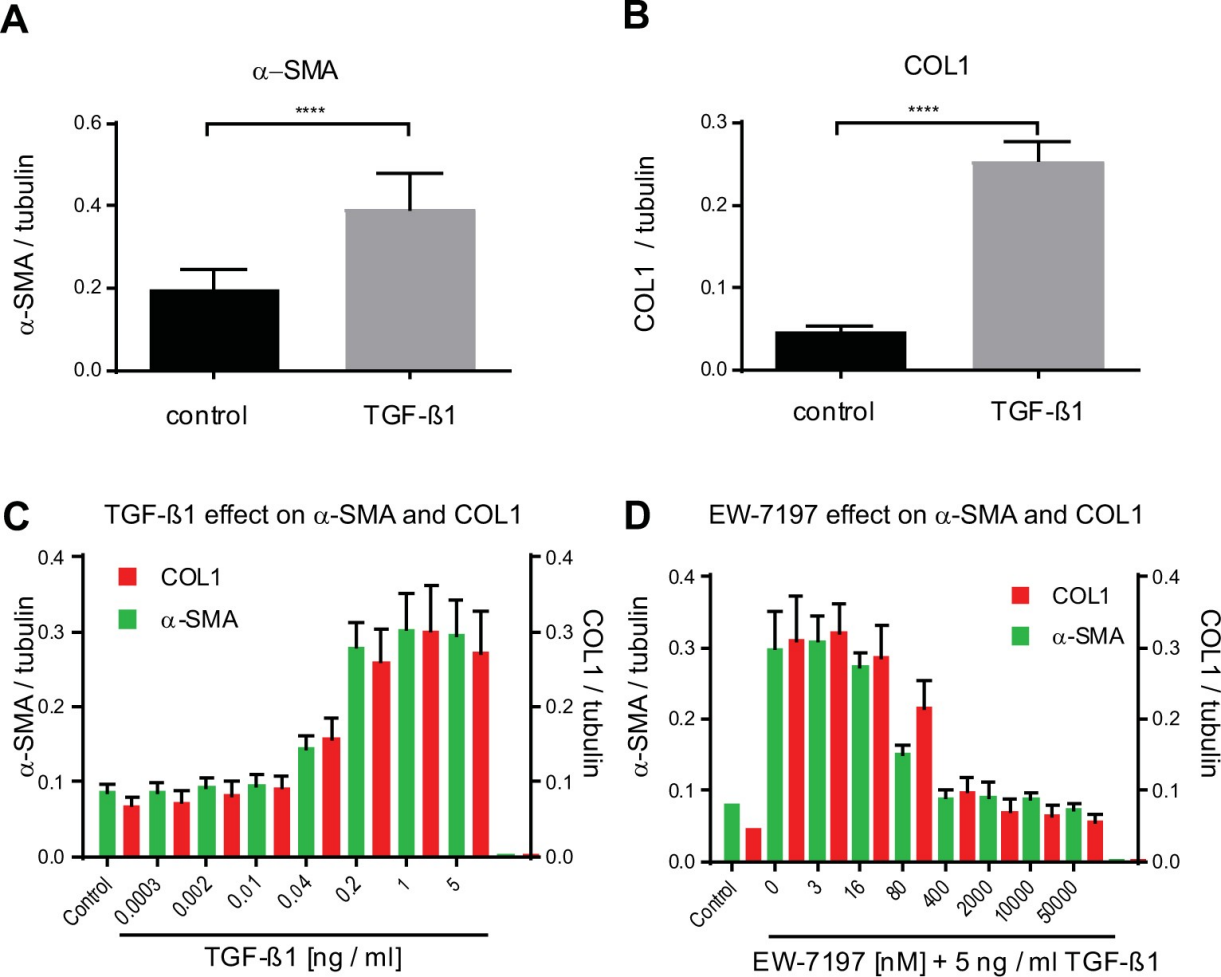
High–throughput–MS / MS quantification of α–SMA, COL1 and tubulin in lysate of NHLF cells. NHLF cells were stimulated with 5 ng / ml TGF–β1 and lysed after 24 h. (A) α–SMA and (B) COL1 were quantified by MS / MS and normalized to tubulin. Data are means and bars show SD of control (black) and TGF–β1–treated (grey) replicates (n = 16). A two–tailed unpaired t test comparing control to TGF–β1–treated is given; **** p < 0.0001. (C) NHLF cells stimulated with increasing concentrations of TGF–β1 were lysed after 48 h and α–SMA, COL1, and tubulin (used for normalization), were quantified from single–wells by MS / MS. (D) NHLF cells were incubated at t = 0 h with dilution series of the ALK5–blocker EW–7197 (0–50,000 nM) and then stimulated with 5 ng / ml TGF–β1 or not (control). Cells were lysed 48 h after TGF–β1 addition and α–SMA and COL1 were quantified. 0.2% DMSO solvent was present in all wells. All analytes were normalized to tubulin. Bars show mean ± SD, n = 8.

As shown by immunoblot (S1 Fig), TGF–β1 increased the ECM proteins COL1 and FN, as well as α–SMA in NHLF. This effect is blocked by the ALK5 inhibitor EW–7197. In order to assess the effect of TGF–β1 on myofibroblast marker proteins, the cells, after they were monitored for impedance for 24 h or 48 h, were lysed and COL1 and α–SMA were quantified using high–throughput MS / MS detection. Tubulin was quantified to normalize for cell numbers. The observed increase in the impedance of fibroblast cell layers in response to 5 ng / ml TGF–β1 was paralleled by a continuous increase over time of both α–SMA and COL1 protein, 24 h (Fig 3A and 3B) and 48 h post TGF–β1 stimulation (Fig 3C). At 48 hours, TGF–β1 increased α–SMA and COL1 protein about 3–fold with an EC_50_ of 0.07 ± 0.01 ng / ml (n = 8) and 0.05 ± 0.01 ng / ml (n = 8), respectively. The ALK5 blocker EW–7197 effectively inhibited the accumulation of α–SMA (IC_50_ = 51 ± 17 nM) and COL1 (IC_50_ = 111 ± 30 nM, n = 8; Fig 3D). Therefore, the potency of TGF–β1 to induce COL1 and α–SMA protein, as detected by MS / MS, was comparable to the potency to induce impedance changes and to the potency of TGF–β1 in the HCA assay (Fig 1C).

To investigate the role of TGF–β1 signaling in our assays, 20 different inhibitors targeting 14 proteins that are associated with TGF–β signaling were investigated and IC_50_ values were determined (Table 1). The compound potency correlated well between the different assay formats (Table 2 and S1 Fig). In addition to the two ALK5 receptor inhibitors, SB525334 and EW–7197, halofuginone, an inhibitor of SMAD–3 phosphorylation [20], very potently and effectively inhibited TGF–β1–induced myofibroblast formation (Tables 1 and 3 and S2 Fig). The two TAK1 inhibitors, 5Z–7–oxozeaenol and salinomycin, as well as inhibitors of c–Jun–N–terminal kinase (JNK) and p38 MAPK, acting downstream of TAK1, were also effective (Table 1). Furthermore, blocking PDK1 / Akt signaling with the inhibitor BX–912 blunted TGF–β1–induced effects. The two ROCK1 / 2 inhibitors GSK–269962 and RKI1447 both inhibited the pro–fibrotic effects of TGF–β1 in both assay systems (HCA and IPQA, Table 1). In summary, these results provide evidence for the critical involvement of canonical ALK5 / SMAD–3–mediated and non–canonical TGF–β1–induced fibroblast–to–myofibroblast transition and the influence of those pathways can be quantified in both assay formats.

**Table 1:** IC_50_ values for small molecule inhibitors of TGF–β1 signaling.

Compound name	Activity	Impedance IC_50_ [nM]	MS / MS α–SMA IC_50_ [nM]	MS / MS COL1 IC_50_ [nM]	HCA IC_50_ [nM]
EW–7197	ALK5 inhibitor	47±25 (n = 7)	51±17 (n = 7)	110±30 (n = 7)	80±30 (n = 7)
SB525334	ALK5 (4) inhibitor	120±25 (n = 2)	90±5 (n = 2)	380±110 (n = 2)	2,500±1,200 (n = 2)
Halofuginone	p–SMAD–3 inhibitor	96±10 (n = 2)	27±4 (n = 2)	27±1 (n = 2)	690±320 (n = 2)
5Z–7–Oxozeaenol	TAK1 inhibitor	250±170 (n = 3)	300±270 (n = 3)	460±270 (n = 3)	2,600±2,500 (n = 2)
Salinomycin	TAK1/p38 pathway inhibitor	1,500±1,500 (n = 2)	290±300 (n = 2)	1,200±1,500 (n = 2)	15,800 (n = 1)
SP600125	JNK1/2/3 inhibitor	48,500±2,100 (n = 2)	38,000±17,000 (n = 2)	36,000±18,000 (n = 2)	>46,000± (n = 2)
L–skepinone	p38 inhibitor	5,000±49 (n = 2)	960±440 (n = 2)	1,500 (n = 1)	23,000 (n = 1)
BX–912	PDK1 inhibitor	7,500±1,600 (n = 3)	8,600±9,400 (n = 3)	8,700±3,000 (n = 2)	17,000±6,700 (n = 2)
GSK–269962	ROCK1/2 inhibitor	1,010±300 (n = 3)	22±1 (n = 2)	3,100±92 (n = 2)	240±270 (n = 2)
RKI 1447	ROCK1/2 inhibitor	8,400±42 (n = 2)	150±22 (n = 2)	12,200±2,400 (n = 2)	4,300±2,300 (n = 2)
BIX 02189	ERK5 inhibitor	6,000 (n = 1)	14,000 (n = 1)	6,500 (n = 1)	22,000±11,000 (n = 2)
XMD 8–92	ERK5 inhibitor	4,400 (n = 1)	2,400 (n = 1)	5,400 (n = 1)	12,000 (n = 1)
FR180204	ERK1/2 inhibitor	50,000 (n = 1)	49,000 (n = 1)	36,000 (n = 1)	>46,000 (n = 1)
PD98059	MEK inhibitor	19,000 (n = 1)	50,000 (n = 1)	>50,000 (n = 1)	>46,000 (n = 1)
Pamapimod	p38 inhibitor	19,000 (n = 1)	34,000 (n = 1)	>50,000 (n = 1)	>46,000 (n = 1)
Ipatasertib	AKT1 / 2 / 3 inhibitor	43,000 (n = 1)	25,000 (n = 1)	>50,000 (n = 1)	22,000 (n = 1)
EHT 1865	RAC inhibitor	14,000 (n = 1)	16,000 (n = 1)	28,000 (n = 1)	not assessed
LY294002	PI3K a/d/b inhibitor	13,000 (n = 1)	27,000 (n = 1)	45,000 (n = 1)	2,400 (n = 1)
IC–87114	PI3K g / d inhibitor	>5,000 (n = 1)	>5,000 (n = 1)	4,900 (n = 1)	44,000 (n = 1)
Csn–B	NR4A1 agonist (nuclear receptor)	>50,000 (n = 1)	>50,000 (n = 1)	>50,000 (n = 1)	39,000 (n = 1)

Values represent mean ± SD; n, number of independent experiments.

**Table 2:** Correlation of IC_50_ values between assay read–outs for 20 compounds inhibiting TGF–β1 signaling.

Assay 1 ^1^	Assay 2	Spearman r	p value
MS / MS α–SMA	MS / MS COL1	0.8558	< 0.0001
Impedance	MS / MS COL1	0.9182	< 0.0001
Impedance	MS / MS α–SMA	0.8984	< 0.0001
MS / MS α–SMA	High–content assay	0.8283	< 0.0001
MS / MS COL1	High–content assay	0.7133	< 0.0005

^1^ IC_50_’s (nM) for assays 1 and 2 were compared.

**Table 3:** IC_50_ values of selected compounds in the standard assay.

Compound name	Nuclear count IC_50_ [nM]	Impedance IC_50_ [nM]^1^	MS / MS α–SMA IC_50_ [nM]	MS / MS COL1 IC_50_ [nM]	HCA IC_50_ [nM]
EW–7197	>25,000 (n = 1)	47 ± 25 (n = 7)	51 ± 17 (n = 7)	110 ± 30 (n = 7)	82 ± 30 (n = 7)
Nintedanib	1,400 (n = 1)	300 ± 110 (n = 2)	500± 120 (n = 2)	1,600 ± 1,300 (n = 3)	760 ± 130 (n = 3)
Halofuginone	>46,000 (n = 1)	96 ± 11 (= 2)	27 ± 4 (n = 2)	27 ± 1 (n = 2)	690 ± 320 (n = 2)
GSK–269962	>50,000 (n = 1)	1,010 ± 300 (n = 3)	22 ± 1 (n = 2)	3,100 ± 92 (n = 2)	240 ± 270 (n = 2)
Alprostadil	>50,000 (n = 2)	0.6 ± 0.1 (n = 3)	1.9 ± 1.6 (n = 3)	5.0 ± 1.4 (n = 3)	8.6 ± 10 (n = 2)
PGE_2_	>2,500 (n = 1)	0.5 ± 0.1 (n = 2)	1.2 ± 1.2 (n = 2)	4.9 ± 4.4 (n = 2)	n.a.
ONO–18c	>50,000 (n = 1)	0.04 ± 0.01 (n = 3)	0.05 ± 0.01 (n = 2)	0.9 ± 0.5 (n = 3)	< 2.5 (n = 2)
ONO–18k	>50,000 (n = 2)	1.3 ± 1.1 (n = 4)	2.6 ± 2.9 (n = 3)	7.6 ± 5.7 (n = 4)	110 ± 150 (n = 2)
Evatanepag	>50,000 (n = 2)	6.7 ± 3 (n = 3)	26 ± 29 (n = 2)	11 (n = 1)	82 ± 110 (n = 2)
Merck–19a	n.a.	10.4 ± 1.1 (n = 2)	108 ± 87 (n = 2)	17.7 ± 7.0 (n = 2)	n.a.
Digoxigenin	19,000 (n = 1)	250 ± 140 (n = 2)	38 ± 6 (n = 2)	85 ± 8 (n = 2)	220 (n = 1)

n.a.: not assessed. Values represent mean ± SD of n independent experiments.

^1^ Impedance data were extracted at t = 20 h post TGF–β1 addition.

### Screening of a library of 1585 approved drugs in the HCA

A library of 1585 approved drugs was screened by HCA at fixed compound concentrations of 10 μM to identify compounds that attenuated TGF–β1–induced myofibroblast formation by greater than 50% over a period of 48 h. Z’ values ranged from 0.8 to 0.9, indicating that the assay was very robust. The potency of those compounds that reduced cell viability by less than 50% was determined in concentration response experiments in the same assays. Of the initially selected 127 hit compounds, 42 had IC_50_ values below 10 μM (S2 Table).

In our assays, nintedanib, one of two currently approved drugs for IPF, inhibited TGF–β1–induced myofibroblast formation in the HCA, inhibited impedance changes, and attenuated α–SMA and COL1 increase in IPQA (Table 3).

Strikingly, 6 of the 10 most potent molecules, ouabain, digoxin, digoxigenin, digitoxigenin, proscillaridin A, and lanatoside C, shared a cardiac glycoside scaffold. These cardiac glycosides very potently prevented TGF–β1–induced myofibroblast formation in the HCA (S2 Table). The cardiac glycoside digoxigenin prevented TGF–β1–induced myofibroblast formation in the HCA (Table 3 and S3 Fig), blocked TGF–β1–induced effects in IPQA, and inhibited α–SMA and COL1 neo–synthesis (Table 3 and S3 Fig). It also reduced cell surface area, as evidenced by the CellMask Orange stain, at concentrations ≥ 1250 nM, and reduced the number of nuclei with IC_50_ 19 μM (n = 1; Table 3) in the HCA.

**Fig 4:**
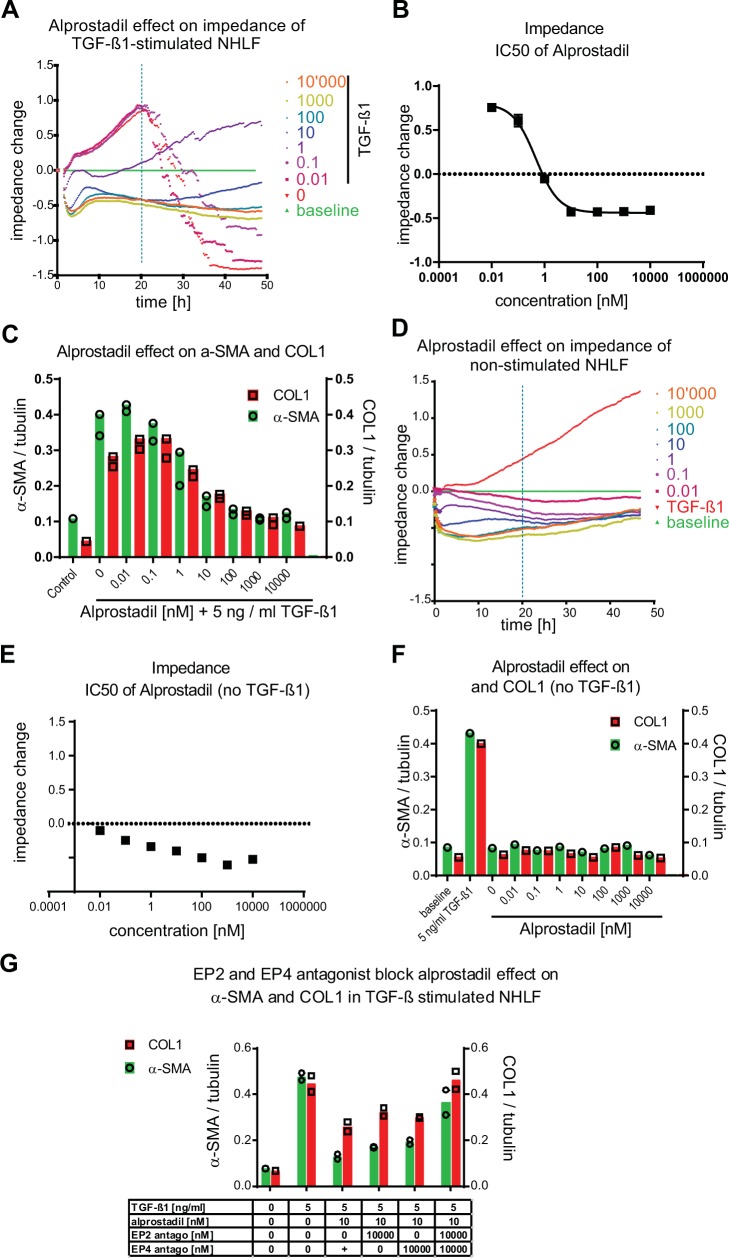
Alprostadil inhibits TGF–β1–induced changes in NHLF by activating both, the EP_2_ and the EP_4_ receptor,without affecting baseline α–SMA and COL1 levels in non–stimulated NHLF. NHLF fibroblasts were incubated with dilution series of alprostadil (0.01–10,000 nM) at t = 0 h and then stimulated (A–C) or not (D–F) with TGF–β1 (5 ng / ml). Non–stimulated NHLF cells (0 ng / ml TGF–β1, 0 nM alprostadil, baseline) and 5 ng / ml TGF–β1–treated (0 nM alprostadil; vehicle) are depicted in green and red, respectively (A, D). Concentration response curves of alprostadil in presence (B) and absence (E) of 5 ng / ml TGF–β1 where then generated with baseline subtracted impedance values at t = 20 h post TGF–β1 addition. (G) At t = 0 h NHLF fibroblasts were incubated with 10,000 nM of the EP_2_ prostaglandin receptor antagonist, the EP_4_ receptor antagonist, and of both the EP_2_ and the EP_4_ receptor antagonists in the absence or in presence of 10 nM alprostadil and 5 ng / ml TGF–β1. At t = 48 h after TGF–β1 (C, G) or vehicle (F) addition the cells were lysed and α–SMA and COL1 were quantified by MS / MS. Bars represent protein data normalized to tubulin. Number of sample (n = 1) is shown in each figure A, D, E, F. Bars represent mean of n = 2 samples in B, C and G.

Among the most potent and fully effective antifibrotic compounds identified, and without decreasing the number of nuclei, alprostadil inhibited TGF–β1–induced fibroblast–to–myofibroblast transition in the HCA (Tables 3 and S2), inhibited TGF–β1–induced impedance changes (Fig 4A and 4B) and inhibited α–SMA and COL1 increases in IPQA (Table 3 and Fig 4C). Alprostadil is a close analog of the prostaglandin receptor agonist prostaglandin E2 (PGE_2_), previously reported as an anti–fibrotic that activates EP_2_ and EP_4_ receptors [21, 22]. In the absence of TGF–β1, alprostadil lowered the impedance of the cell layer below the baseline (Fig 4D and 4E) but otherwise had no effect on basal α–SMA and COL1 expression (Fig 4F). PGE_2_ reproduced the observed effects of alprostadil in our assays with similar potency and efficacy (S4 Fig). NHLF fibroblasts, in the presence of 10 nM alprostadil, were resistant to differentiation in response to 5 ng / ml TGF–β1 (S5 Fig). Inhibition of alprostadil action on both the EP_2_ and EP_4_ receptors with the EP_2_ receptor antagonist PF–04418948 [11] combined with the EP_4_ antagonist MK–2894 [12] abrogated the inhibitory effect and allowed myofibroblast differentiation to progress. The inhibitory effect of alprostadil was only partially reversed when either EP_2_ or EP_4_ receptor alone was blocked (Figs 4G and S5). Hence, the EP receptor agonist alprostadil exerts its anti–fibrotic effect by activating EP_2_ and EP_4_ receptors.

### EP_2_ and EP_4_ receptor–specific agonists prevent fibroblast–to–myofibroblast transition

To determine if selective activation of the EP_2_ receptor is sufficient for anti–fibrotic effect, and if so, if it is mediated through cAMP, EP_2_ receptor selective agonists were tested. The EP_2_ receptor–selective agonist evatanepag (also known as CP–533536) [10], the EP_2_ receptor–selective Gαs–signaling biased agonist compound ONO–18k, and the EP_2_ receptor–selective un–biased agonist ONO–18c [9] were used.

In order to confirm their functional selectivity and signaling mode, these molecules were characterized in EP_2_ and EP_4_ receptor cAMP and β–arrestin assays (S3 Table). ONO–18c efficiently recruited β–arrestin (E_max_ = 160%) and very potently and efficiently induced cAMP production in EP_2_ receptor recombinant HEK293 cells (EC_50_ < 0.01 nM; E_max_ = 98%). As expected for a Gα–protein–biased selective EP_2_ receptor agonist compound ONO–18k was less effective in recruiting β–arrestin in EP_2_ receptor–expressing recombinant cells (E_max_ = 32%) but increased cAMP production in EP_2_ receptor–expressing recombinant HEK293 cells (EC_50_ = 0.03 nM, E_max_ 99%). Furthermore, ONO–18k was inactive on EP_4_ receptor–expressing HEK293 cells (EC_50_ > 10,000 nM; E_max_ = 24%) [9]. Evatanepag displayed selectivity and signaling that was comparable to ONO–18k (S3 Table).

**Fig 5:**
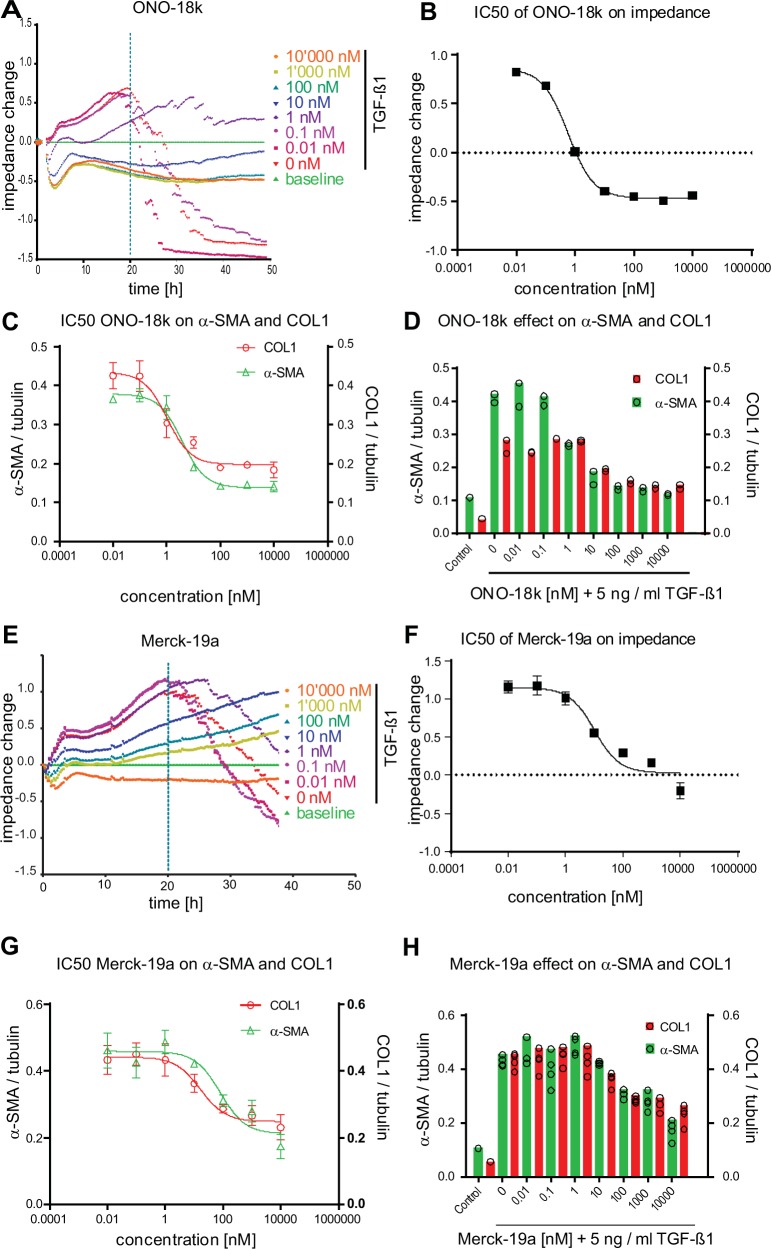
EP_2_ and EP_4_ receptor–selective agonists inhibit TGF–β1–induced fibroblast–to–myofibroblast transition of NHLF. NHLF cells were incubated with dilution series (0.01–10,000 nM) of the Gαs–biased EP_2_ receptor agonist ONO–18k (–A) or the EP_4_–receptor selective agonist Merck–19a (E) and then stimulated with 5 ng / ml TGF–β1. Impedance responses were monitored for 48 h. Impedance traces of non–stimulated NHLF cells (0 ng / ml TGF–β1; baseline; green traces) and NHLF cells stimulated with 5ng / ml TGF–β1 in the absence of compound (0 nM agonist; vehicle, red traces) are depicted. Concentration response curves of ONO–18k (B) or Merck–19a (F) in presence of 5 ng / ml TGF–β1 where then generated with baseline (0 ng / ml TGF–β1) subtracted impedance values at t = 20 h post TGF–β1 addition (mean ± SD, n = 2). At t = 48 h after TGF–β1 addition the cells were lysed and α–SMA and COL1 were quantified by MS / MS. (C, G) Concentration response curves and (D, H) bar charts of ONO–18k or Merck–19a dilution series in presence of 5 ng / ml TGF–β1 where generated from α–SMA and COL1 protein data normalized to tubulin (mean ± SD, n = 2).

In NHLF cells, ONO–18c inhibited TGF–β1–induced impedance changes and attenuated increases in the myofibroblast marker proteins α–SMA and COL1 in IPQA (S6 Fig and Table 3). ONO–18k dose–dependently inhibited TGF–β1–induced impedance changes, as well as α–SMA and COL1 increases in IPQA (Fig 5 and Table 3). These data demonstrate that cAMP elevation, induced by selective activation of EP_2_ receptors, is sufficient to prevent TGF–β1–mediated fibroblast–to–myofibroblast transition in NHLF. To determine if selective activation of the EP_4_ receptor is equally sufficient for anti–fibrotic effect, the EP_4_ receptor selective agonists Merck–19a (CAY10598) [13] was used. Compound Merck–19a was shown to selectively bind to the EP_4_ receptor with a K_i_ value of 1.2 nM [13]. In our assays, Merck–19a inhibited TGF–β1–induced impedance changes and blocked increases in α–SMA and COL1 in IPQA (Fig 5 and Table 3).

### De–differentiation of myofibroblasts to fibroblasts

**Fig 6:**
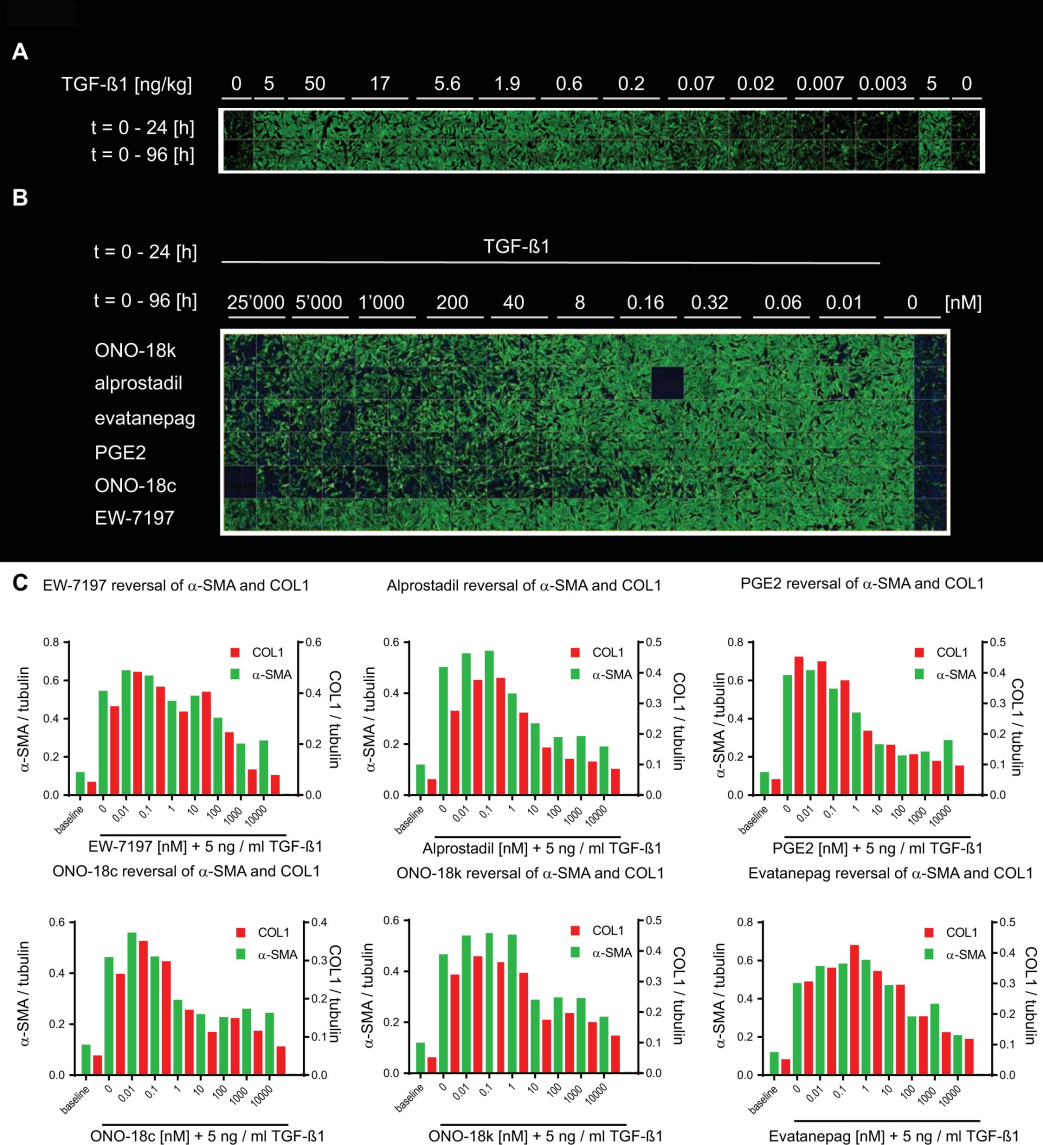
Reversal of TGF–β1–induced NHLF myofibroblast phenotype. (A) After starvation NHLF cells were stimulated with a dilution series of TGF–β1 (0.003–50 ng / ml) either for 24 h (after which cells were washed and cultured for further 72 h without TGF–β1, top row) or for the entire duration of 96 h (bottom row). Cells were fixed and immunostained to detect the myofibroblast markers FN and α–SMA by high–content confocal microscopy. Shown are the green channel images representing α–SMA. The final assay concentration of DMSO was 0.6% in all wells. (B) Confocal images of NHLF cells that were differentiated for 24 h with 5 ng / ml TGF–β1 into myofibroblasts. The cells were then washed 3 times to remove TGF–β1 and incubated for 72 h with increasing concentrations (0–10,000 nM) of the agonists ONO–18k, alprostadil, evatanepag, PGE_2_ and ONO–18c, as well as with the ALK5 blocker EW–7197. (C) Data represent α–SMA (green bars) and COL1 (red bars) quantified by MS / MS and normalized to tubulin, at t = 96 h after TGF–β1 addition. Number of sample (n = 1) is shown in each figure.

To identify compounds with potential to revert differentiated myofibroblasts to fibroblasts the assay procedures were modified. After 24 h starvation, NHLF cells were stimulated with 5 ng / ml TGF–β1 for 24 h. At t = 24 h TGF–β1 was removed and either vehicle (DMSO) or compounds were added and incubated with the cells for a further 72 h. HCA readouts showed that NHLF fibroblasts stimulated with TGF–β1 for 24 h and further cultured for 72 h in the absence of TGF–β1 remained myofibroblast–like and they were indistinguishable from myofibroblasts that were continuously exposed to TGF–β1 for 96 hours (Fig 6A). In this setting, the effect of the ALK5 blocker EW–7197 appeared limited in the HCA (Fig 6B and Table 4 and on impedance at t = 20 h (IC_50_ > 10,000 nM, Table 4), but potent on impedance at t = 72 h post TGF–β1 (IC_50_ = 47 ± 1 nM, n = 2; S7 Fig) and on α–SMA and COL1 in the IPQA (Table 4 and Fig 6C). Alprostadil, PGE_2_, ONO–18c, ONO–18k, and evatanepag, all reversed myofibroblasts into cells with fibroblast–like appearance (Fig 6 and Table 4) in the HCA.

**Table 4:** IC_50_ values of selected compounds in the NHLF myofibroblast reversal assay.

Compound name	Nuclear count IC_50_ [nM]	Impedance IC_50_ [nM]^1^	MS / MS α–SMA IC_50_ [nM]	MS / MS COL1 IC_50_ [nM]	HCA IC_50_ [nM]
EW–7197	>25,000 (n = 1)	>10,000 (n = 2)	73 ± 31 (n = 2)	82 ± 30 (n = 2)	18,000 (n = 1)
Nintedanib	16,000 (n = 1)	>10,000 (n = 1)	>10,000 (n = 1)	626 (n = 1)	6,900 (n = 1)
Halofuginone	1,400 (n = 1)	>1000 (n = 2)	102 ± 87 (n = 2)	116 ± 81 (n = 2)	120 (n = 1)
GSK–269962	n.a.	>10,000 (n = 1)	35 (n = 1)	537 (n = 1)	n.a.
Alprostadil	>25,000 (n = 1)	0.9 ± 0.2 (n = 2)	1 ± 0.0 (n = 2)	1.5 ± 0.7 (n = 2)	280 (n = 1)
PGE_2_	>25,000 (n = 1)	0.7 (n = 1)	0.8 (n = 1)	0.4 (n = 1)	145 (n = 1)
ONO–18c	>25,000 (n = 1)	0.1 ± 0.02 (n = 2)	0.14 ± 0.09 (n = 2)	0.2 ± 0.14 (n = 2)	20 (n = 1)
ONO–18k	>25,000 (n = 1)	2 (n = 1)	3 (n = 1)	2 (n = 1)	5,500 (n = 1)
Evatanepag	>25,000 (n = 1)	14 (n = 1)	16 (n = 1)	24 (n = 1)	2,300 (n = 1)
Digoxigenin	2,700 (n = 1)	140 (n = 1)	48 (n = 1)	54 (n = 1)	76 (n = 1)

n.a.: not assessed. Mean ± SD of n independent experiments are shown.

^1^Impedance data were extracted at t = 20 h post TGF–β1 addition.

**Fig 7:**
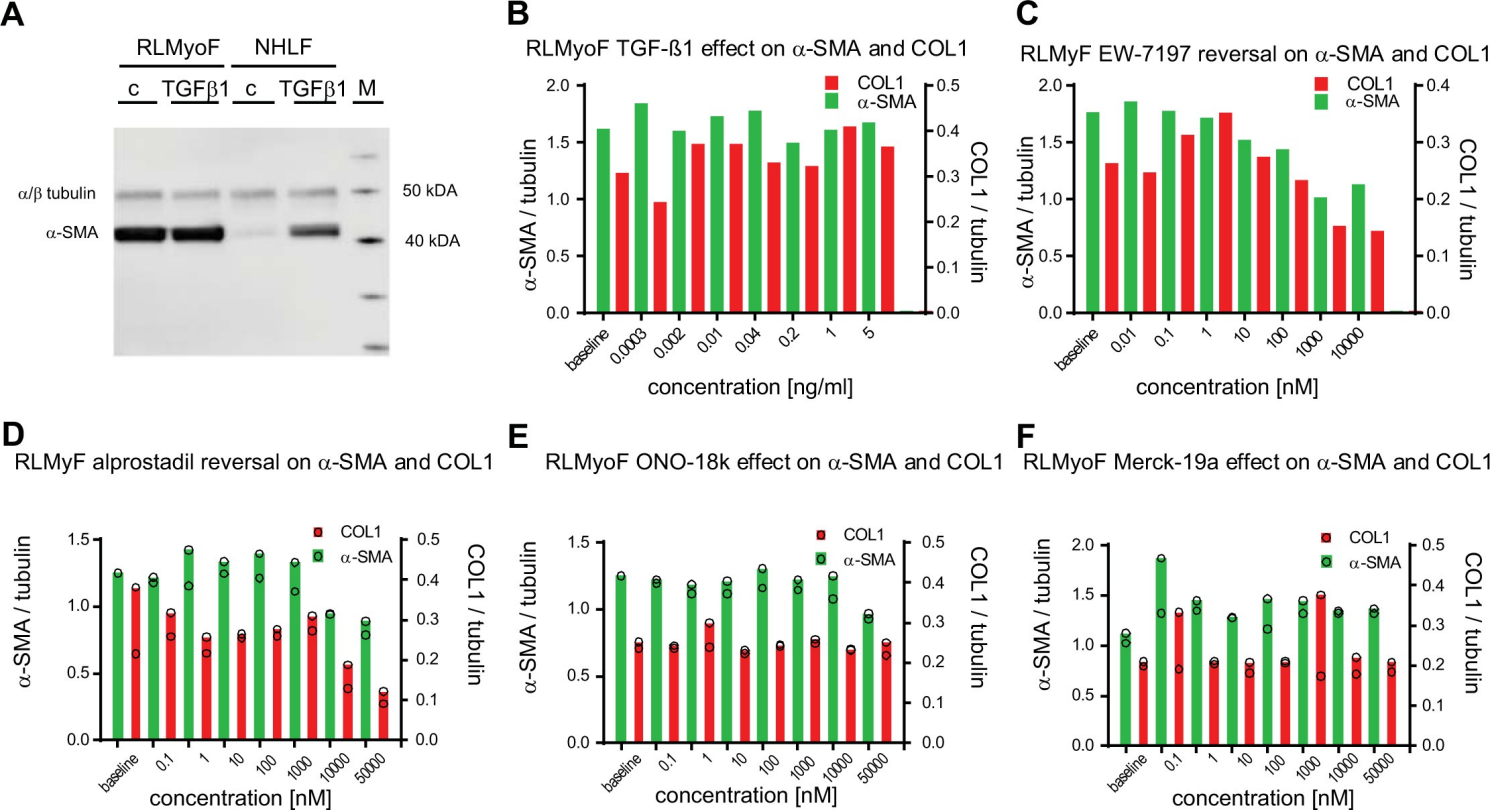
Effects on α–SMA and COL1 levels in primary myofibroblasts isolated from fibrotic lungs of bleomycin–instilled aged Wistar rats. (A) Primary NHLF and myofibroblasts isolated from fibrotic rat lung middle lobes (RLMyoF) 28 days after bleomycin instillation were serum starved for 24 h and stimulated either with 5 ng / ml TGF–β1 (TGF–β) or with the appropriate vehicle control (c) for 48 h. α–SMA (42 kDa) and α / β–tubulin (50 kDa) were quantified by immunoblot analysis. The protein molecular weight marker (M) was run in parallel to estimate protein size. (B) RLMyoF were starved for 24 h, stimulated with a dilution series of TGF–β1 for 24 h, washed, cultured in the absence of TGF–β1 for further 72 h, and lysed to quantify α–SMA, COL1 and tubulin from single wells by MS / MS detection. (C–F) Non–stimulated RLMyoF were starved for 24 h and then exposed to increasing concentrations of compound for further 72 h. Cells were lysed and α–SMA (green bars) and COL1 (red bars) were quantified by MS / MS to assess the effect of the ALK5 inhibitor EW–7197 (C), alprostadil (D), and the EP_2_ and EP_4_ receptor selective agonists ONO–18k (E), and Merck–19a (F), respectively. All analytes were normalized to tubulin. 0.1% DMSO solvent was present in all wells. Number of sample (n = 1) in (B, C) and (n = 2) in (D–F).

Next, we tested whether the reversal to a more fibroblast–like phenotype is also possible for primary myofibroblasts that were isolated from fibrotic lungs. To this end, lung fibrosis was induced in 20–month–old Wistar rats by bleomycin instillation [23]. Myofibroblasts that were isolated from the fibrotic lungs 4 weeks after bleomycin instillation had high α–SMA expression, as detected by immunoblot analysis (Fig 7A). The IPQA allowed quantification of human and rat tubulin, α–SMA, and COL1 with the same specificity and confirmed strong and persistent baseline α–SMA and COL1 expression in the isolated rat lung myofibroblasts (RLMyoF) in the absence of TGF–β1 stimulation. The levels of α–SMA, normalized to tubulin, in TGF–β1 unstimulated RLMyoF (Fig 7) were even higher than those in NHLF cells treated with 5 ng / ml TGF–β1 for 48 h (Fig 6). Addition of TGF–β1 did not lead to any further increase in α–SMA and COL1 (Fig 7B and S4 Table) in RLMyoF, which suggests that RLMyoF represent fully differentiated myofibroblasts. As observed for the TGF–β1–induced NHLF myofibroblasts, exposure of RLMyoF to the ALK5 inhibitor EW–7197 led to a pronounced decrease in both, α–SMA and COL1, with IC_50_'s of 68 nM and 126 nM (n = 1; Fig 7C), respectively. Alprostadil was ineffective at pharmacological concentrations relevant to activate the EP_2_ and EP_4_ receptors but decreased α–SMA and COL1, with IC_50_'s of 8450 ± 1610 nM (n = 2) and 2380 ± 2730 nM (n = 2; Fig 7D). The two EP_2_ receptor–specific agonists ONO–18c and ONO–18k and the EP_4_ receptor–specific agonist Merck–19a had no effect on α–SMA and COL1 in differentiated RLMyoF (Fig 7).

## Discussion

In pulmonary fibrosis, myofibroblasts synthesize and deposit large amounts of ECM and, through the formation of cellular stress fibers, contribute to lung stiffening and the progressive loss of lung function. We have developed novel cellular assays that allow screening of a large number of compounds for their ability to prevent or reverse myofibroblast differentiation. The degree of myofibroblast differentiation was quantified using a HCA in combination with a secondary IPQA assay that allowed detection of impedance changes multiplexed with quantification of α–SMA and COL1 from lysed cells. We screened 1585 approved drugs and identified, amongst other compounds, alprostadil as a highly potent and effective inhibitor of TGF–β1–mediated NHLF–to–myofibroblast differentiation.

The fact that nintedanib was effective in our assays underscores the relevance of our tests and underlines the validity of myofibroblasts as a therapeutic target for pulmonary fibrosis. Our findings are consistent with published data showing that nintedanib inhibited TGF–β2 (10 ng / ml)–induced differentiation of primary human lung fibroblasts (from IPF patients) to myofibroblast in an α–smooth muscle actin mRNA expression assay [24].

**Fig 8:**
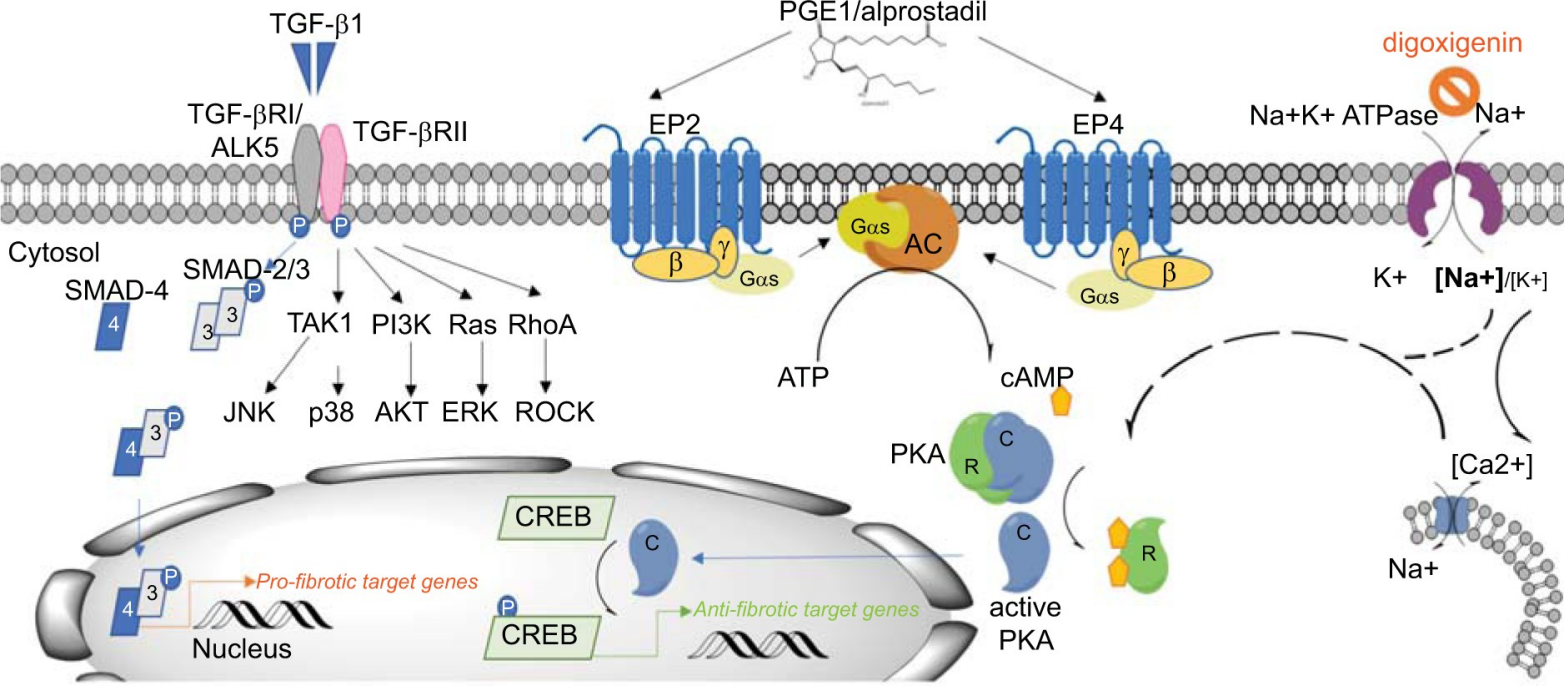
Summary binding of active TGF–β dimer to TGF–β receptor type II leads to the formation of a stable complex with the type I/ activin receptor–like kinase (ALK5) receptor and its transphosphorylation and activation. In the case of the canonical TGF–β signaling pathway, this results in the activation of SMAD family transcription factors SMAD–2 and –3 which, upon phosphorylation, form complexes with SMAD–4 and translocate to the nucleus, where they act in transcriptional complexes as regulators of pro–fibrotic down–stream genes [6]. In our assay, TGF–β1 signaling involved ALK5 and SMAD3, but also all three mitogen–activated protein kinase (MAPK) pathways: e,g, the extracellular signal–regulated kinase (ERK), p38 MAPK, and c–Jun–N–terminal kinase (JNK) pathways. In addition, TGF–β1 effect was mediated by PI3 kinase / Akt and Rho GTPase pathways. Binding of alprostadil to the G–protein coupled receptors EP_2_ and EP_4_, which are linked to G–αs proteins, induced the adenylate cyclase–mediated cAMP second messenger formation from ATP. It is well established that cAMP acts by activating protein kinase A (PKA) [28, 29], resulting in dissociation of the regulatory (R) and the catalytic (C) subunits of the kinase. The catalytic subunits translocate to the nucleus and initiate the activation of the cAMP response element binding (CREB) transcription factor, and, as a consequence, transcription of downstream genes. Cardiac glycosides concentration–dependently increased the intracellular [Na^+^]_i_/[K^+^]_i_ ratio, which led to an increase in COX2 expression and PKA activation and decreased α–SMA, COL1 and FN [30]. To which extent this effect was mediated by an increase in intracellular Ca^2+^ concentration through activation of the reverse mode of an Na^+^/Ca^2+^ exchanger warrants further clarification.

We applied TGF–β1 to NHLF cells to induce myofibroblast differentiation as it is well known to be a critical mediator of fibrogenesis [6]. Indeed, both, the HCA and the IPQA assay indicated that TGF–β1 induced a robust and sustained myofibroblast differentiation of NHLF. As expected, the two ALK5 receptor inhibitors, SB525334 and EW–7197, as well as the alkaloid drug halofuginone, which interferes with SMAD–3 phosphorylation downstream of TGF–β1 receptor activation [20, 25], very potently and effectively inhibited TGF–β1–induced myofibroblast differentiation. TGF–β1–mediated effects were also blocked by compounds targeting non–canonical SMAD–independent signaling intermediates that include p38 MAPK, JNK, PI3K / Akt, and Rho GTPase. Our findings, which are in line with previous reports [6, 26, 27], suggest that effective myofibroblast differentiation engages non–canonical signaling pathways in addition to canonical TGF–β1 / SMAD3 signaling and that these pathways either converge at common downstream nodes or exhibit significant crosstalk (Fig 8).

Alprostadil binds the prostaglandin PGE_2_ receptor subtypes EP_1_ to EP_4_. Decreased production of prostaglandins is predictive of, and contributes to, fibrotic lung disease [29]. *Ptger2*^*–/–*^knock–out mice lacking a functional copy of EP_2_ receptor exhibited a more severe fibrotic response to bleomycin instillation compared to wild–type mice [31]. Elevating PGE_2_ levels in serum by preventively administering PGE_2_ via surgically implanted minipumps or in the lung by knocking out the plasminogen activation inhibitor–1 (*Pai–1*) gene blunted bleomycin–induced lung fibrosis [32–35]. In lung fibroblasts, PGE_2_–mediated EP_2_ receptor activation inhibited proliferation [36] and collagen expression via cAMP / PKA–mediated effects [21, 22, 36]. Furthermore, PGE_2_ reversed the phenotype of TGF–β1–induced myofibroblasts [22, 37, 38].

Our results with the G–protein signaling–biased EP_2_ receptor–specific agonists show that activation of the EP_2_ receptor, via Gαs signaling, is sufficient to block and reverse TGF–β1 –induced NHLF myofibroblasts (Figs 5 and 6). The same effect was achieved with forskolin, an activator of cAMP synthesis (S7 Fig). Furthermore, the EP_4_ receptor–selective compound Merck–19a inhibited TGF–β1–mediated myofibroblast differentiation (Fig 5). This implies that in TGF–β1–differentiated NHLF EP_2_ and EP_4_ receptor expression is sufficiently high to stimulate the synthesis of cAMP, which inhibits TGF–β1 activity.

We first confirmed that alprostadil, and its close analog PGE_2_, by stimulating EP_2_ and EP_4_ receptors, increase cellular cAMP content in recombinant HEK293 cells (S3 Table). In addition, both alprostadil and PGE_2_ prevented and even reversed TGF–β1–induced NHLF myofibroblasts. However, in presence of a combination of both, an EP_2_ and an EP_4_ receptor–specific antagonist, alprostadil lost its anti–fibrotic effect. Inhibition of either EP_2_ or EP_4_ receptor alone was insufficient to block TGF–β1–induced effects (Fig 4G). These data show that: (i) NHLF cells express, in addition to the EP_2_ receptor, also the EP_4_ receptor and (ii) the anti–fibrotic effect of alprostadil was mediated through the EP_2_ receptor when the EP_4_ receptor was blocked, and through the EP_4_ receptor when the EP_2_ receptor alone was blocked.

It is well established that PGE_2_, and its close analog alprostadil, activate the prostanoid receptors EP_1_, EP_2_, EP_3_ and EP_4_ with high affinity [39]. Whereas activation of the Gαs–protein–dependent EP_2_ and EP_4_ receptors, leads to an increase in cytosolic cAMP levels, signaling via EP_1_ receptor, coupled to Gαq, increases intracellular [Ca^2+^], while signaling via the EP_3_ receptor, coupled to Gαi, reduces cellular cAMP. Hence, activation of EP_1_ or EP_3_ is not expected to contribute to the anti–fibrotic effect of alprostadil. In contrary, EP_3_ receptor activation is expected to show opposite effects to EP_2_ / EP_4_ receptor activation.

In RLMyoF alprostadil, which is able to activate cAMP via both, the EP_2_ and the EP_4_ receptors, induced a concentration–dependent impedance reduction (EC_50_ = 1.5 ± 0.8 nM, n = 2) down to the baseline (S8 Fig). However, in RLMyoF the corresponding decrease of α–SMA and COL1 only occurred with a huge potency shift (Fig 7) compared with its effect on TGF–β1–induced myofibroblasts (Figs 6 and S7) and at much higher concentrations than are necessary to activate its receptors EP_2_ and EP_4_ (S3 Table). While the EP_2_ selective agonist did not trigger an impedance response in RLMyoF (S8 Fig), the EP_4_ receptor–selective agonist Merck–19 partially decreased the impedance in RLMyoF (EC_50_ = 83 nM, n = 1; S8 Fig), indicative of a signal event, while the lack of impedance response indicates absence of receptor activation by the selective EP_2_ receptor agonist. Hence, our impedance data indicate that in RLMyoF the individual EP_2_ and EP_4_ receptor density is low. In view of this, it may come as no surprise that neither selective activation of the EP_2_ receptor nor of the EP_4_ receptor led to a reduction of α–SMA or COL1.

The use of the EP_2_ and EP_4_ receptor selective agonists revealed differences between TGF–β1–induced myofibroblasts and RLMyoF. It cannot be excluded that the compound effects observed in TGF–β1–differentiated myofibroblasts cannot generally be extrapolated to RLMyoF. Therefore, the extent to which either TGF–β1–induced myofibroblasts or RLMyoF reflect human disease requires further investigation. In our assays, blocking canonical TGF–β1–signaling, either with the ALK5 blocker EW–7197 or with halofuginone, reversed pre–differentiated NHLF–derived myofibroblasts. Indicating that continued ALK5 / SMAD–3–mediated signaling was a pre–requisite to keep TGF–β1–induced myofibroblasts in a differentiated state. To test whether fully differentiated myofibroblasts that were not stimulated with TGF–β1 *in vitro* also have the capacity to de–differentiate into fibroblasts and to exclude the possibility of interfering residual TGF–β1 due to incomplete washing, RLMyoF were isolated from fibrotic lungs of bleomycin–instilled Wistar rats. The isolated RLMoyF showed persistent and high basal levels of α–SMA and COL1, which were not further increased in response to TGF–β1 stimulation, underscoring the fact that these RLMyoF represent fully differentiated myofibroblasts. As already observed with the NHLF–myofibroblasts, treatment of RLMyoF with the ALK5 inhibitor EW–7197 led to a pronounced decrease in both, α–SMA and COL1 in IPQA, indicating that continuous ALK5–mediated signaling is indeed required to maintain the fibrotic RLMyoF in their differentiated myofibroblast state. Several factors have been reported to reverse fibroblast–to–myofibroblast differentiation *in vitro* [37, 40–44]. However, blocking TGF–β signaling did not de–differentiate chicken embryo dermal myofibroblasts [45, 46], TGF–β1–induced human fetal IMR90 myofibroblasts [44] and rat cardiac myofibroblasts, but de–differentiated mechanical stress–induced cardiac myofibroblasts [47]. Here, both, RLMyoF and NHLF myofibroblasts, when cultured in the absence of any added TGF–β1, remained differentiated, and NHLF cultured under the same starved conditions did not differentiate into myofibroblasts, thus our data are consistent with the hypothesis that lung myofibroblasts synthesize and secrete an autocrine signaling molecule to maintain ALK5–mediated myofibroblast differentiation.

Formation of adhesive contacts was critical for myofibroblast differentiation of IMR90 cells. When IMR90 cells in suspension were treated with TGF–β1 no myofibroblast differentiation occurred despite effective ligation of TGF–β1 with its receptors, as evidenced by downstream SMAD–2 phosphorylation [26]. Disassembly of focal contacts and stress fibers leads to relaxation of the cytoskeleton [48]. Several molecules that were active in our myofibroblast reversal assays triggered an immediate and concentration–dependent impedance drop with a nadir 2–3 h after addition of the substance, compatible with relaxation of the cytoskeleton. This was observed not only for the compounds which increase intracellular cAMP levels, but also for the ALK5 blocker EW–7197, as well as for the ROCK1/2 inhibitor GSK–269962 (S8 Fig). The formation of focal adhesions involves activation of members of the small Ras GTPase family (Rho A, Rac, or CDC42) [29] known to activate ROCK effector kinases implicated in stress fiber formation [49]. In our assays inhibition of ROCK1/2 effectively blocked myofibroblast differentiation and reversed differentiated myofibroblasts. Hence, ROCK1/2 function might prevent relaxation–induced de–differentiation and, thus, contribute to the maintenance of a stably differentiated myofibroblast phenotype.

In our pilot screen 6 cardiac glycoside compounds effectively prevented TGF–β1–induced myofibroblast differentiation at low concentrations. For all cardiac glycosides the calculated IC_50_ values for inhibition of myofibroblast formation were in the low nanomolar range and, hence, close to published potencies for inhibiting Na^+^ / K^+^–ATPase activity [50, 51]. Thus, our data confirm and extend the published findings that both ouabain and digoxin prevented myofibroblast differentiation of lung fibroblasts in response to TGF–β1 [30]. In addition, the representative cardiac glycoside digoxigenin fully reversed the observed TGF–β1–induced NHLF myofibroblast phenotypes in all our assays. The data described by La *et al*. support a mechanism of action by which cardiac glycosides induce cyclooxygenase–2 (COX–2) expression and PKA activation, which might lead to increased prostaglandin synthesis and decreased Rho activation in fibroblasts [30]. This is consistent with our findings that both prostaglandins and ROCK inhibitors effectively inhibited TGF–β1–induced NHLF differentiation.

Despite an overall good correlation between the assays and their different readouts, compounds, when measured in the HCA, often appeared to be less potent compared to IPQA. The reason for this is not clear, but could be rooted in the fundamentally different methodologies used to assess the phenotype of the cells. It is therefore possible that compounds have remained undetected in our screens despite their anti–fibrotic potential.

Here we describe two novel high–throughput screening assays and we identified molecules that were able to inhibit and / or revert myofibroblast differentiation. In particular, the EP_2_ / EP_4_ receptor non–selective agonist alprostadil and the cardiac glycoside digoxigenin effectively reversed myofibroblast attributes in the absence of exogenous TGF–β1 as did the ALK5 inhibitor EW–7197. These results support the hypothesis that autocrine TGF–β–signaling maintains the differentiated state of myofibroblasts. Our screen identified molecules with strong anti–fibrotic properties and the potential to serve as starting points for future therapies for IPF patients and those with other fibrotic disease.

## Supporting information

S1 Table Complete feature list and their weighted relevance for the differentiating capability of the analysis pipeline. (PDF)

S2 Table The 42 hits identified by screening 1’585 approved drugs with the HCA. (PDF)

S3 Table Selectivity and potency of EP_2_ receptor agonists. (PDF)

S4 Table Effect of TGF–β1 on the α–SMA / tubulin and COL1 / tubulin ratios in NHLF and RLMyoF. (PDF)

S1 Fig Correlation of IC_50_ values between assay read–outs for 20 compounds inhibiting TGF–β1 signaling. IC50 data was log transformed using Graph Pad Prism and linear regression was performed on the logarithms. (A) Plot of impedance and α–SMA data. (B) Plot of impedance and COL1 data. (C) Plot of HCA and α–SMA data. (D) Plot of HCA and COL1 data. (E) Plot of α–SMA and COL1 data. (F) Primary NHLF were serum starved for 24 h and stimulated either with 5 ng / ml TGF–β1 (TGF–β) or with the appropriate vehicle control, in presence or absence of the ALK5 blocker EW-7197 or alprostadil for 48 h. α–SMA (42 kDa, Sigma # A2547), α / β–tubulin (50 kDa, CellSignaling # 2148), collagen 1 α 1 (139 kDa, Aviva Systems Biology # OAMA03716) and fibronectin (~250 kDa, Santa Cruz Biotech # sc-6952) were visualized by immunoblot analysis. The protein molecular weight marker (Invitrogen # LC5925) was run in parallel to estimate protein size. IPQA data were generated from NHLF donor 1 with 5 ng / ml TGF–β1 in presence compound and / or 0.5% DMSO (vehicle) for 48 h; n = 1. HCA data were generated from NHLF donor 2 treated with 5 ng / ml TGF–β1 in presence compound and / or 0.5% DMSO (vehicle) for 48 h; mean of n = 2. R square (r^**2**^) and p value of linear regression are indicated. P < 0.05 was considered significant.
(PDF)

S2 Fig Halofuginone inhibits TGF–β1–induced changes in NHLF. Shown are impedance traces of non–stimulated NHLF cells (0 ng / ml TGF–β1; baseline, green), NHLF cells stimulated with 5ng / ml TGF–β1 in the absence of compound (0 nM compound, red), and NHLF cells exposed to dilutions series of halofuginone (0.01–10,000 nM). Due to cytotoxicity the impedance data corresponding to 10,000 nM halofuginone (marked with an asterisk) were excluded for the IC50 calculation (A). Concentration response curves of halofuginone in presence of 5 ng / ml TGF–β1 where then generated with baseline (0 ng / ml TGF–β1) subtracted impedance values at t = 20 h post TGF–β1 addition (B). At t = 48 h after TGF–β1 addition the cells were lysed and α–SMA (C) and COL1 (E) were quantified by MS / MS. Bars represent protein data normalized to tubulin. Concentration response curves of halofuginone in presence of 5 ng / ml TGF–β1 where then generated with the normalized α–SMA (D) and COL1 (F) data. One of two very similar experiments is shown.
(PDF)

S3 Fig Digoxigenin inhibits TGF–β1–induced myofibroblast differentiation of NHLF. The effect of TGF–β1 (5 ng / ml) is inhibited by increasing concentrations (0.016 nM– 25’000 nM) of the cardiac glycoside digoxigenin as captured by high–content confocal microscopy 48 h after TGF–β1 stimulation. Nuclei are stained with DAPI, α–SMA, FN and the cytosol with anti–α–SMA IgG, anti–FN IgG and CellMaskTM Orange, respectively (A). Impedance recordings of non–stimulated NHLF cells (0 ng / ml TGF–β1; baseline, green), NHLF cells stimulated with 5ng / ml TGF–β1 in the absence of compound (0 nM compound, red), and NHLF cells exposed to dilutions series of digoxigenin (0.004–4,000 nM) (B). Concentration response curves of digoxigenin in presence of 5 ng / ml TGF–β1 where then generated with baseline (0 ng / ml TGF–β1) subtracted impedance values at t = 20 h post TGF–β1 addition. One of two very similar experiments is shown. (C). At t = 48 h after TGF–β1 addition the cells were lysed and α–SMA and COL1 were quantified by MS / MS. Bars represent mean (n = 2) of protein amount normalized to tubulin (D).
(PDF)

S4 Fig PGE_2_ prevents TGF–β1–induced changes in NHLF. Impedance recordings of non–stimulated NHLF cells (0 ng / ml TGF–β1; baseline, green), NHLF cells stimulated with 5ng / ml TGF–β1 in the absence of compound (0 nM compound, red), and NHLF cells exposed to dilutions series of PGE2 (0.01–10,000 nM) (A). Concentration response curves of PGE2 in presence of 5 ng / ml TGF–β1 where generated with baseline (0 ng / ml TGF–β1) subtracted impedance values at t = 20 h post TGF–β1 addition (B). At t = 48 h after TGF–β1 addition the cells were lysed and α–SMA and COL1 were quantified by MS / MS. Bars represent protein data obtained from a single well normalized to tubulin from NHLF cells incubated with dilution series of PGE2 followed by stimulation with 5 ng / ml TGF–β1 (C). Concentration response curves in presence of 5 ng / ml TGF–β1 where generated with the normalized α–SMA and COL1 data (D).
(PDF)

S5 Fig Alprostadil activates the EP_2_ and the EP_4_ receptor to inhibit TGF–β1–induced changes in NHLF. (A) Impedance changes of NHLF cells incubated with 10 nM alprostadil and 5 ng / ml TGF–β1, exposed to dilution series of an EP_**2**_ receptor antagonist, and EP_**4**_ receptor antagonist or the combination of the EP_**2**_ and the EP_**4**_ antagonist, were exported at t = 20 h and plotted against the compound concentration to generate concentration response curves. (B) At t = 0 h NHLF fibroblasts were stimulated with 5 ng / ml TGF–β1 and incubated with dilution series (0.01–10,000 nM) of alprostadil (B), or the EP_**2**_ prostaglandin receptor antagonist (C), the EP_**4**_ receptor antagonist (D), and of both the EP2 and the EP_**4**_ receptor antagonists (E) in the absence (B) or in presence (C–E) of 10 nM alprostadil. At t = 48 h after TGF–β1 addition the cells were lysed and α–SMA and COL1 were quantified by MS / MS. Bars represent protein data normalized to tubulin. Data of one representative experiment is shown in B. Data shown in C–E represent mean ± SD (n = 2).
(PDF)

S6 Fig Non–prostanoid EP_2_ receptor selective agonists prevent the appearance of TGF–β1–induced myofibroblast features in NHLF cells. (A) Impedance recordings of NHLF cells exposed to dilution series of the selective EP_**2**_ receptor agonists ONO–18c, ONO–18k, and evatanepag (0.01–10,000 nM), followed by stimulation with TGF–β1 (5ng / ml). Impedance traces of non–stimulated vehicle–treated NHLF cells (0 ng / ml TGF–β1; baseline) and of NHLF fibroblasts stimulated with 5ng / ml TGF–β1 (0 nM agonist; vehicle) are highlighted in green and red color, respectively. Impedance changes at t = 20 h were exported and plotted against the agonist concentration for IC50 calculation. (B) At t = 48 h after TGF–β1 addition the cells were lysed and α–SMA and COL1 were quantified by MS / MS. Bars represent protein data normalized to tubulin. (C) Concentration response curves were generated for alprostadil and the selective EP2 agonists ONO–18c, ONO–18k, and evatanepag of normalized α–SMA (top row) and COL1 (bottom row) from NHLF fibroblasts stimulated with 5 ng / ml TGF–β1.
(PDF)

S7 Fig Reversal of TGF–β1–induced myofibroblast phenotype of NHLF cells. NHLF cells were differentiated for 24 h with 5 ng / ml TGF–β1 into myofibroblasts. The cells were then washed 3 times to remove TGF–β1 and incubated with increasing concentrations (0–25,000 nM) of the cardiac glycosides digoxigenin, digoxin and digitoxigenin (A), with halofuginione or with nintedanib / BIBF1120 (B) for 72 h in starvation medium. Cells were fixed and immunostained to detect the myofibroblast markers FN and α–SMA (green) by high–content confocal microscopy. (C) Impedance recordings of NHLF cells pre–stimulated for 24 h with 5 ng / ml TGF–β1 treated with dilution series (0.01–10,000 nM) of the ALK5 blocker EW–7197, alprostadil, PGE2, of the selective EP2 agonists ONO–18c, ONO–18k and evatanepag, of the adenylyl cyclase activator forskolin, as well as of the antifibrotic halofuginone and the cardiac glycoside digoxigenin. Impedance traces of non–stimulated NHLF cells (0 ng / ml TGF–β1; baseline) and of NHLF fibroblasts stimulated with 5ng / ml TGF–β1 (0 nM agonist; vehicle) are highlighted in green and red color, respectively. IC50 concentrations were calculated from normalized MS / MS data to quantify the effect of increasing concentrations (0.01–10,000 nM) of EW–7197, alprostadil, PGE2, ONO–18c, ONO–18k, evatanepag, forskolin, halofuginone, as well as digoxigenin, on accumulated α–SMA (D) and COL1 (E) in NHLF fibroblasts pre–stimulated with 5 ng / ml TGF–β1 for 24h. Representative experiments are shown.
(PDF)

S8 Fig Effects on impedance in primary myofibroblasts isolated from fibrotic lungs of bleomycin–instilled aged Wistar rats. (A-C) Impedance recordings of non–stimulated RLMyoF that were starved for 24 h and then exposed for further 72 h to dilution series (0.01–10,000 nM) of the selective EP2 receptor agonists ONO–18c (A, D), alprostadil (B, E), or the ROCK1/2 inhibitor GSK-269962 (C, F) are shown. Impedance traces of non–stimulated vehicle–treated NHLF cells (0 ng / ml TGF–β1; baseline) and of NHLF fibroblasts stimulated with 5ng / ml TGF–β1 (0 nM agonist; vehicle) are shown for comparison in green and red color, respectively. (D-F) Impedance changes at t **=** 3 h and t **=** 20 h were exported and plotted against the agonist concentration for IC50 calculation.
(PDF)
