# Supplementary material for: Novel high–throughput myofibroblast assays identify agonists with therapeutic potential in pulmonary fibrosis that act via EP2 and EP4 receptors
Source: PLoS One. 2018 Nov 28;13(11):e0207872. doi: 10.1371/journal.pone.0207872 (PMC6261607; doi:10.1371/journal.pone.0207872)
Supplement: S1 Table — (PDF) [file pone.0207872.s001.pdf]

Supplementary Material

S1 Table. Complete feature list and their weighted relevance for the differentiating capability of the analysis pipeline

Rank	Feature	WeightSum	Weights_0
1	Granularity_1_CorrFN_2	10.01331338	10.01331338
2	Granularity_1_CorrACT_1	8.77621353	8.77621353
3	Granularity_3_CorrACT_1	8.583349788	8.583349788
4	Granularity_3_CorrFN_2	5.340851793	5.340851793
5	Granularity_2_CorrACT_1	5.250555548	5.250555548
6	Granularity_6_CorrFN_2	4.713900903	4.713900903
7	Intensity_MeanIntensityEdge_CorrFN_1	4.151370198	4.151370198
8	Granularity_4_CorrACT_1	3.675790181	3.675790181
9	Granularity_16_CorrFN_2	3.452529283	3.452529283
10	Granularity_2_CorrFN_2	3.438477474	3.438477474
11	Neighbors_SecondClosestDistance_Expanded_0	3.148662	3.148662
12	Granularity_4_CorrFN_2	3.033126438	3.033126438
13	Granularity_5_CorrACT_1	3.025140137	3.025140137
14	Texture_SumAverage_CorrFN_3_0_1	2.876709315	2.876709315
15	Granularity_5_CorrFN_2	2.824770281	2.824770281
16	Granularity_10_CorrFN_2	2.703537969	2.703537969
17	Granularity_8_CorrFN_2	2.59733461	2.59733461
18	Granularity_9_CorrFN_2	2.53432678	2.53432678
19	AreaShape_Solidity_1	2.529930649	2.529930649
20	Intensity_MinIntensity_CorrACT_1	2.450605699	2.450605699
21	Intensity_MADIntensity_CorrACT_1	1.894352612	1.894352612
22	Granularity_7_CorrFN_2	1.880843441	1.880843441
23	RadialDistribution_FracAtD_CorrFN_1of4_2	1.834428564	1.834428564
24	Intensity_MeanIntensityEdge_CorrFN_2	1.75336316	1.75336316
25	RadialDistribution_RadialCV_CorrFN_4of4_2	1.597057877	1.597057877

26	AreaShape_FormFactor_2	1.501139599	1.501139599
27	Texture_AngularSecondMoment_CorrACT_3_0_1	1.487525271	1.487525271
28	Texture_InfoMeas1_CorrFN_3_0_1	1.395888009	1.395888009
29	Granularity_14_CorrFN_2	1.35649062	1.35649062
30	Texture_Gabor_CorrFN_3_2	1.344524649	1.344524649
31	Texture_InverseDifferenceMoment_CorrDAPI_3_0_0	1.269087887	1.269087887
32	Intensity_IntegratedIntensityEdge_CorrFN_1	1.122772504	1.122772504
33	Texture_InverseDifferenceMoment_CorrFN_3_0_0	1.086230661	1.086230661
34	Granularity_15_CorrFN_2	1.033514619	1.033514619
35	Texture_DifferenceEntropy_CorrACT_3_0_1	0.957948696	0.957948696
36	Granularity_7_CorrACT_1	0.934765146	0.934765146
37	Granularity_16_CorrACT_1	0.918618585	0.918618585
38	RadialDistribution_MeanFrac_CorrFN_3of4_2	0.887773492	0.887773492
39	RadialDistribution_RadialCV_CorrFN_1of4_2	0.872478237	0.872478237
40	AreaShape_Zernike_7_3_0	0.855899651	0.855899651
41	Texture_DifferenceVariance_CorrACT_3_0_1	0.811849555	0.811849555
42	AreaShape_MinorAxisLength_1	0.799998489	0.799998489
43	Intensity_MaxIntensityEdge_CorrACT_1	0.755680065	0.755680065
44	Intensity_MinIntensityEdge_CorrFN_2	0.73103481	0.73103481
45	RadialDistribution_RadialCV_CorrACT_2of4_1	0.73023483	0.73023483
46	Granularity_12_CorrACT_1	0.72434858	0.72434858
47	Texture_InfoMeas2_CorrDAPI_3_0_1	0.712379706	0.712379706
48	Texture_DifferenceVariance_CorrACT_3_0_0	0.704637156	0.704637156
49	Texture_Gabor_CorrACT_3_1	0.637964129	0.637964129
50	Intensity_MaxIntensityEdge_CorrACT_2	0.627479633	0.627479633
51	Texture_DifferenceVariance_CorrFN_3_0_0	0.626404123	0.626404123
52	Intensity_IntegratedIntensity_CorrACT_2	0.610972326	0.610972326
53	Texture_InverseDifferenceMoment_CorrFN_3_0_1	0.610790892	0.610790892
54	Granularity_13_CorrFN_2	0.601998678	0.601998678

55	RadialDistribution_RadialCV_CorrFN_1of4_1	0.597243935	0.597243935
56	AreaShape_MajorAxisLength_2	0.594952952	0.594952952
57	Texture_InfoMeas1_CorrACT_3_0_1	0.58013592	0.58013592
58	Texture_InfoMeas1_CorrFN_3_0_0	0.571444887	0.571444887
59	AreaShape_Zernike_7_5_1	0.558046168	0.558046168
60	Granularity_11_CorrFN_2	0.557850509	0.557850509
61	Granularity_15_CorrACT_1	0.557623947	0.557623947
62	Texture_AngularSecondMoment_CorrDAPI_3_0_1	0.543461866	0.543461866
63	Intensity_IntegratedIntensityEdge_CorrFN_2	0.534751958	0.534751958
64	RadialDistribution_FracAtD_CorrFN_2of4_1	0.510743852	0.510743852
65	Granularity_6_CorrACT_1	0.504067477	0.504067477
66	AreaShape_Zernike_9_9_1	0.494023186	0.494023186
67	Texture_Correlation_CorrDAPI_3_0_2	0.493682233	0.493682233
68	Texture_DifferenceEntropy_CorrDAPI_3_0_0	0.493594196	0.493594196
69	Texture_Gabor_CorrACT_3_0	0.477984151	0.477984151
70	Granularity_9_CorrACT_1	0.476260128	0.476260128
71	Texture_InfoMeas1_CorrDAPI_3_0_1	0.472507582	0.472507582
72	Texture_Variance_CorrACT_3_0_1	0.467134699	0.467134699
73	Texture_SumVariance_CorrFN_3_0_0	0.434800099	0.434800099
74	Texture_InverseDifferenceMoment_CorrACT_3_0_0	0.433763572	0.433763572
75	Texture_Gabor_CorrDAPI_3_2	0.413979998	0.413979998
76	Intensity_IntegratedIntensity_CorrFN_2	0.413358048	0.413358048
77	AreaShape_Zernike_2_2_0	0.412016655	0.412016655
78	Texture_InfoMeas1_CorrFN_3_0_2	0.411224074	0.411224074
79	Texture_Variance_CorrFN_3_0_1	0.404369108	0.404369108
80	Intensity_UpperQuartileIntensity_CorrFN_2	0.403875338	0.403875338
81	AreaShape_Zernike_5_3_2	0.40261658	0.40261658
82	Texture_Gabor_CorrDAPI_3_1	0.40183852	0.40183852
83	Intensity_IntegratedIntensityEdge_CorrACT_2	0.392272362	0.392272362
84	Texture_Variance_CorrDAPI_3_0_1	0.388572903	0.388572903

85	Texture_Gabor_CorrACT_3_2	0.38811043	0.38811043
86	Texture_InfoMeas2_CorrACT_3_0_1	0.376651162	0.376651162
87	AreaShape_Zernike_1_1_2	0.375348071	0.375348071
88	Intensity_MinIntensityEdge_CorrACT_1	0.372607158	0.372607158
89	AreaShape_FormFactor_1	0.369352323	0.369352323
90	AreaShape_Zernike_1_1_0	0.360522231	0.360522231
91	AreaShape_Eccentricity_0	0.358851453	0.358851453
92	Texture_SumAverage_CorrDAPI_3_0_1	0.353173165	0.353173165
93	AreaShape_Solidity_2	0.352109834	0.352109834
94	AreaShape_Zernike_7_1_1	0.351767001	0.351767001
95	Intensity_MinIntensityEdge_CorrFN_1	0.349182628	0.349182628
96	Texture_InfoMeas2_CorrACT_3_0_2	0.341562147	0.341562147
97	Texture_Correlation_CorrDAPI_3_0_0	0.339057655	0.339057655
98	AreaShape_Zernike_9_5_2	0.336531479	0.336531479
99	AreaShape_Perimeter_2	0.326366333	0.326366333
100	Texture_SumVariance_CorrACT_3_0_1	0.317467554	0.317467554
101	AreaShape_Zernike_4_4_1	0.310640159	0.310640159
102	RadialDistribution_RadialCV_CorrFN_2of4_2	0.310406037	0.310406037
103	RadialDistribution_RadialCV_CorrACT_3of4_1	0.310136869	0.310136869
104	RadialDistribution_FracAtD_CorrFN_3of4_1	0.308129374	0.308129374
105	Granularity_13_CorrACT_1	0.300777243	0.300777243
106	RadialDistribution_FracAtD_CorrFN_3of4_2	0.292868407	0.292868407
107	Texture_AngularSecondMoment_CorrDAPI_3_0_2	0.285682964	0.285682964
108	RadialDistribution_RadialCV_CorrACT_4of4_2	0.271923329	0.271923329
109	RadialDistribution_MeanFrac_CorrACT_3of4_2	0.265164967	0.265164967
110	Intensity_StdIntensity_CorrFN_1	0.261451471	0.261451471
111	Texture_Entropy_CorrDAPI_3_0_0	0.259420809	0.259420809
112	AreaShape_Zernike_6_0_1	0.25840834	0.25840834
113	Texture_DifferenceEntropy_CorrFN_3_0_1	0.256767108	0.256767108
114	Intensity_MADIntensity_CorrACT_2	0.256649849	0.256649849
115	RadialDistribution_MeanFrac_CorrACT_3of4_1	0.253102423	0.253102423

116	Texture_DifferenceVariance_CorrDAPI_3_0_0	0.250564088	0.250564088
117	AreaShape_MajorAxisLength_1	0.24714089	0.24714089
118	Intensity_MeanIntensity_CorrFN_2	0.244760684	0.244760684
119	Texture_DifferenceEntropy_CorrACT_3_0_2	0.241938361	0.241938361
120	Texture_SumVariance_CorrDAPI_3_0_0	0.238078351	0.238078351
121	Texture_Contrast_CorrFN_3_0_0	0.238038621	0.238038621
122	Intensity_MeanIntensityEdge_CorrACT_2	0.230215651	0.230215651
123	Intensity_MassDisplacement_CorrACT_1	0.227904539	0.227904539
124	Intensity_MeanIntensityEdge_CorrACT_1	0.224816704	0.224816704
125	RadialDistribution_RadialCV_CorrFN_3of4_2	0.223742674	0.223742674
126	Texture_Variance_CorrACT_3_0_0	0.221679492	0.221679492
127	AreaShape_Compactness_2	0.22013309	0.22013309
128	AreaShape_Zernike_6_6_0	0.218806372	0.218806372
129	Texture_InfoMeas1_CorrDAPI_3_0_0	0.213401817	0.213401817
130	Intensity_MassDisplacement_CorrACT_2	0.211672454	0.211672454
131	RadialDistribution_RadialCV_CorrACT_2of4_2	0.209974023	0.209974023
132	Intensity_MaxIntensityEdge_CorrFN_2	0.206613419	0.206613419
133	AreaShape_MedianRadius_1	0.205857689	0.205857689
134	AreaShape_Zernike_8_2_1	0.202160248	0.202160248
135	AreaShape_Zernike_9_7_1	0.201334857	0.201334857
136	Texture_AngularSecondMoment_CorrFN_3_0_0	0.201087621	0.201087621
137	AreaShape_Area_2	0.199331925	0.199331925
138	RadialDistribution_FracAtD_CorrACT_3of4_1	0.198592439	0.198592439
139	RadialDistribution_RadialCV_CorrFN_4of4_1	0.197562965	0.197562965
140	Texture_Correlation_CorrFN_3_0_0	0.194973537	0.194973537
141	Texture_DifferenceEntropy_CorrFN_3_0_0	0.194824587	0.194824587
142	Intensity_StdIntensityEdge_CorrFN_2	0.19470226	0.19470226
143	AreaShape_Zernike_9_3_0	0.192155265	0.192155265
144	Texture_InfoMeas1_CorrACT_3_0_0	0.191168082	0.191168082
145	Texture_Entropy_CorrFN_3_0_2	0.190698813	0.190698813
146	AreaShape_Zernike_4_4_2	0.190172972	0.190172972

147	RadialDistribution_FracAtD_CorrACT_3of4_2	0.189335568	0.189335568
148	Texture_DifferenceVariance_CorrDAPI_3_0_2	0.188974221	0.188974221
149	Texture_SumEntropy_CorrACT_3_0_0	0.185043357	0.185043357
150	AreaShape_Eccentricity_1	0.184182098	0.184182098
151	Granularity_8_CorrACT_1	0.184082916	0.184082916
152	AreaShape_Zernike_4_0_0	0.182435146	0.182435146
153	AreaShape_MaxFeretDiameter_1	0.181991653	0.181991653
154	Intensity_MaxIntensityEdge_CorrFN_1	0.179956751	0.179956751
155	AreaShape_Zernike_2_0_1	0.172260317	0.172260317
156	Intensity_StdIntensityEdge_CorrACT_1	0.17033879	0.17033879
157	AreaShape_Zernike_6_2_1	0.170186601	0.170186601
158	Intensity_MaxIntensity_CorrFN_1	0.166391875	0.166391875
159	Texture_InfoMeas2_CorrFN_3_0_1	0.164969889	0.164969889
160	AreaShape_Extent_1	0.16398657	0.16398657
161	RadialDistribution_FracAtD_CorrFN_2of4_2	0.162830099	0.162830099
162	Intensity_MinIntensity_CorrACT_2	0.159400245	0.159400245
163	Intensity_MassDisplacement_CorrFN_2	0.159084811	0.159084811
164	Texture_InverseDifferenceMoment_CorrACT_3_0_1	0.158956713	0.158956713
165	AreaShape_Zernike_3_3_2	0.158547821	0.158547821
166	Texture_Correlation_CorrFN_3_0_1	0.158188484	0.158188484
167	Texture_Contrast_CorrDAPI_3_0_1	0.157528326	0.157528326
168	AreaShape_Zernike_7_3_2	0.157425549	0.157425549
169	AreaShape_Zernike_8_8_0	0.156604209	0.156604209
170	Texture_SumAverage_CorrDAPI_3_0_0	0.154670116	0.154670116
171	Texture_SumAverage_CorrDAPI_3_0_2	0.152673621	0.152673621
172	AreaShape_Compactness_0	0.151515666	0.151515666
173	AreaShape_Zernike_8_2_2	0.151106962	0.151106962
174	AreaShape_Zernike_3_1_2	0.149390356	0.149390356
175	Texture_Entropy_CorrFN_3_0_0	0.149158945	0.149158945
176	Granularity_11_CorrACT_1	0.14739877	0.14739877
177	AreaShape_MinFeretDiameter_1	0.147317788	0.147317788

178	AreaShape_Zernike_7_7_0	0.146081138	0.146081138
179	Texture_SumAverage_CorrFN_3_0_2	0.145164192	0.145164192
180	Texture_Contrast_CorrDAPI_3_0_0	0.144693634	0.144693634
181	Intensity_MaxIntensity_CorrACT_1	0.143995808	0.143995808
182	AreaShape_Zernike_7_7_1	0.143410129	0.143410129
183	Texture_DifferenceVariance_CorrFN_3_0_2	0.140592424	0.140592424
184	Texture_InfoMeas2_CorrDAPI_3_0_2	0.140493047	0.140493047
185	Intensity_MassDisplacement_CorrFN_1	0.139793669	0.139793669
186	AreaShape_FormFactor_0	0.136699889	0.136699889
187	AreaShape_Zernike_5_5_0	0.1361727	0.1361727
188	Intensity_IntegratedIntensity_CorrACT_1	0.135938316	0.135938316
189	RadialDistribution_RadialCV_CorrFN_2of4_1	0.134731484	0.134731484
190	AreaShape_MedianRadius_2	0.134336287	0.134336287
191	Granularity_14_CorrACT_1	0.133265023	0.133265023
192	Texture_SumVariance_CorrDAPI_3_0_1	0.12644638	0.12644638
193	RadialDistribution_RadialCV_CorrACT_1of4_2	0.122915093	0.122915093
194	RadialDistribution_MeanFrac_CorrACT_4of4_2	0.121478166	0.121478166
195	Texture_InfoMeas2_CorrFN_3_0_0	0.11808821	0.11808821
196	AreaShape_Zernike_4_4_0	0.117069049	0.117069049
197	Granularity_10_CorrACT_1	0.115542472	0.115542472
198	Intensity_UpperQuartileIntensity_CorrFN_1	0.113622604	0.113622604
199	Neighbors_NumberOfNeighbors_Expanded_0	0.11203779	0.11203779
200	AreaShape_Zernike_9_9_0	0.111801837	0.111801837
201	Texture_SumEntropy_CorrDAPI_3_0_0	0.111413884	0.111413884
202	Texture_Gabor_CorrFN_3_0	0.110927778	0.110927778
203	Texture_Gabor_CorrDAPI_3_0	0.110647388	0.110647388
204	RadialDistribution_MeanFrac_CorrFN_3of4_1	0.108888107	0.108888107
205	AreaShape_Zernike_5_5_1	0.108260725	0.108260725
206	AreaShape_MedianRadius_0	0.107760226	0.107760226
207	Texture_InverseDifferenceMoment_CorrDAPI_3_0_1	0.107560543	0.107560543

208	Texture_AngularSecondMoment_CorrACT_3_0_2	0.106405979	0.106405979
209	Texture_Contrast_CorrFN_3_0_1	0.105988684	0.105988684
210	Texture_SumEntropy_CorrDAPI_3_0_2	0.1054513	0.1054513
211	Texture_InfoMeas1_CorrACT_3_0_2	0.10429728	0.10429728
212	Intensity_MADIntensity_CorrFN_1	0.104061383	0.104061383
213	AreaShape_Zernike_0_0_1	0.103336424	0.103336424
214	AreaShape_MinorAxisLength_0	0.102786607	0.102786607
215	AreaShape_Zernike_8_0_2	0.10169128	0.10169128
216	AreaShape_Zernike_6_6_2	0.101493888	0.101493888
217	RadialDistribution_MeanFrac_CorrFN_2of4_1	0.099668526	0.099668526
218	Intensity_StdIntensityEdge_CorrFN_1	0.098155551	0.098155551
219	Texture_SumEntropy_CorrDAPI_3_0_1	0.096693568	0.096693568
220	AreaShape_Zernike_2_2_1	0.095120409	0.095120409
221	AreaShape_Zernike_8_0_0	0.092508732	0.092508732
222	Y	0.091462977	0.091462977
223	Texture_Variance_CorrDAPI_3_0_0	0.090655828	0.090655828
224	Intensity_LowerQuartileIntensity_CorrFN_2	0.088070721	0.088070721
225	AreaShape_MaxFeretDiameter_2	0.087552268	0.087552268
226	Texture_InverseDifferenceMoment_CorrFN_3_0_2	0.087528957	0.087528957
227	X	0.087162885	0.087162885
228	AreaShape_Zernike_5_3_0	0.081428744	0.081428744
229	AreaShape_Extent_2	0.078533746	0.078533746
230	AreaShape_Zernike_9_1_1	0.07818973	0.07818973
231	AreaShape_Zernike_9_1_0	0.077405024	0.077405024
232	AreaShape_Zernike_6_2_2	0.077254524	0.077254524
233	AreaShape_Zernike_7_5_2	0.077236039	0.077236039
234	Intensity_MedianIntensity_CorrACT_1	0.07688111	0.07688111
235	AreaShape_Zernike_0_0_2	0.076387147	0.076387147
236	Texture_SumAverage_CorrFN_3_0_0	0.075843794	0.075843794
237	Texture_DifferenceVariance_CorrDAPI_3_0_1	0.075560477	0.075560477

238	AreaShape_Zernike_8_0_1	0.074041281	0.074041281
239	RadialDistribution_MeanFrac_CorrACT_1of4_1	0.071598459	0.071598459
240	AreaShape_Zernike_5_1_2	0.068631172	0.068631172
241	AreaShape_MaximumRadius_2	0.06842179	0.06842179
242	Texture_AngularSecondMoment_CorrFN_3_0_2	0.066443279	0.066443279
243	Intensity_MinIntensity_CorrFN_1	0.065916044	0.065916044
244	Intensity_StdIntensity_CorrACT_1	0.064912864	0.064912864
245	Texture_Entropy_CorrACT_3_0_2	0.062394234	0.062394234
246	AreaShape_Zernike_6_6_1	0.061755178	0.061755178
247	AreaShape_Zernike_7_1_0	0.061549299	0.061549299
248	AreaShape_Zernike_2_0_0	0.060553445	0.060553445
249	AreaShape_Zernike_8_6_2	0.060315215	0.060315215
250	RadialDistribution_RadialCV_CorrACT_3of4_2	0.059510039	0.059510039
251	AreaShape_Zernike_2_2_2	0.058621583	0.058621583
252	Texture_SumEntropy_CorrFN_3_0_1	0.057844388	0.057844388
253	Texture_DifferenceVariance_CorrACT_3_0_2	0.057838167	0.057838167
254	RadialDistribution_FracAtD_CorrACT_2of4_1	0.056519031	0.056519031
255	AreaShape_Zernike_9_1_2	0.056305402	0.056305402
256	AreaShape_Zernike_9_9_2	0.0557882	0.0557882
257	Texture_Correlation_CorrFN_3_0_2	0.055766007	0.055766007
258	Texture_SumVariance_CorrACT_3_0_0	0.055438295	0.055438295
259	AreaShape_Zernike_4_2_0	0.054765079	0.054765079
260	Texture_Contrast_CorrDAPI_3_0_2	0.054738017	0.054738017
261	Texture_Contrast_CorrACT_3_0_0	0.054666988	0.054666988
262	Intensity_LowerQuartileIntensity_CorrACT_1	0.054549901	0.054549901
263	Texture_AngularSecondMoment_CorrACT_3_0_0	0.054302718	0.054302718
264	Texture_Entropy_CorrDAPI_3_0_2	0.05402575	0.05402575
265	AreaShape_MeanRadius_1	0.053919555	0.053919555
266	Texture_SumEntropy_CorrFN_3_0_0	0.053845026	0.053845026
267	AreaShape_Zernike_8_6_1	0.053298114	0.053298114
268	Intensity_UpperQuartileIntensity_CorrACT_1	0.051430289	0.051430289

269	Intensity_MaxIntensity_CorrACT_2	0.050522246	0.050522246
270	AreaShape_Zernike_6_4_2	0.049434372	0.049434372
271	AreaShape_Zernike_3_1_0	0.049190263	0.049190263
272	Texture_Correlation_CorrACT_3_0_0	0.04769721	0.04769721
273	RadialDistribution_MeanFrac_CorrACT_2of4_2	0.046414262	0.046414262
274	AreaShape_Zernike_7_3_1	0.046064272	0.046064272
275	Intensity_StdIntensity_CorrACT_2	0.045201127	0.045201127
276	AreaShape_Zernike_6_0_0	0.04506589	0.04506589
277	AreaShape_Eccentricity_2	0.043297687	0.043297687
278	RadialDistribution_RadialCV_CorrACT_4of4_1	0.04268633	0.04268633
279	Texture_SumEntropy_CorrACT_3_0_1	0.042149793	0.042149793
280	Texture_InverseDifferenceMoment_CorrDAPI_3_0_2	0.042008293	0.042008293
281	Texture_Variance_CorrACT_3_0_2	0.041827475	0.041827475
282	AreaShape_MinFeretDiameter_0	0.039534979	0.039534979
283	AreaShape_Extent_0	0.039370838	0.039370838
284	Intensity_MinIntensityEdge_CorrACT_2	0.039290524	0.039290524
285	RadialDistribution_FracAtD_CorrACT_1of4_2	0.038838731	0.038838731
286	RadialDistribution_FracAtD_CorrACT_4of4_2	0.038703559	0.038703559
287	Intensity_LowerQuartileIntensity_CorrFN_1	0.038424528	0.038424528
288	Texture_AngularSecondMoment_CorrDAPI_3_0_0	0.038180106	0.038180106
289	AreaShape_Compactness_1	0.03748692	0.03748692
290	AreaShape_Zernike_6_0_2	0.037212828	0.037212828
291	AreaShape_Zernike_9_7_0	0.03658205	0.03658205
292	RadialDistribution_FracAtD_CorrACT_2of4_2	0.036361147	0.036361147
293	RadialDistribution_FracAtD_CorrACT_4of4_1	0.035661552	0.035661552
294	Texture_SumEntropy_CorrFN_3_0_2	0.035425104	0.035425104
295	AreaShape_Zernike_7_5_0	0.035342814	0.035342814
296	AreaShape_MeanRadius_2	0.035140144	0.035140144
297	Intensity_IntegratedIntensityEdge_CorrACT_1	0.034373624	0.034373624
298	Texture_Entropy_CorrACT_3_0_1	0.033742086	0.033742086

299	AreaShape_MaximumRadius_0	0.031085848	0.031085848
300	Texture_DifferenceEntropy_CorrACT_3_0_0	0.029636418	0.029636418
301	AreaShape_Zernike_0_0_0	0.029291801	0.029291801
302	RadialDistribution_MeanFrac_CorrACT_2of4_1	0.02834218	0.02834218
303	Intensity_MaxIntensity_CorrFN_2	0.027703499	0.027703499
304	AreaShape_Zernike_4_2_2	0.027580011	0.027580011
305	Granularity_12_CorrFN_2	0.025861795	0.025861795
306	Intensity_LowerQuartileIntensity_CorrACT_2	0.025836106	0.025836106
307	Texture_InfoMeas2_CorrFN_3_0_2	0.024810177	0.024810177
308	RadialDistribution_MeanFrac_CorrACT_4of4_1	0.024215003	0.024215003
309	AreaShape_Zernike_5_5_2	0.023409923	0.023409923
310	AreaShape_Zernike_8_4_2	0.022886445	0.022886445
311	RadialDistribution_FracAtD_CorrFN_4of4_2	0.022618545	0.022618545
312	AreaShape_Orientation_2	0.022391096	0.022391096
313	AreaShape_Zernike_8_2_0	0.021513391	0.021513391
314	Intensity_MeanIntensity_CorrACT_1	0.021154441	0.021154441
315	AreaShape_Zernike_9_3_2	0.020713199	0.020713199
316	Intensity_IntegratedIntensity_CorrFN_1	0.020368526	0.020368526
317	Texture_AngularSecondMoment_CorrFN_3_0_1	0.019541604	0.019541604
318	Intensity_MeanIntensity_CorrACT_2	0.018523581	0.018523581
319	Texture_SumVariance_CorrFN_3_0_1	0.017541259	0.017541259
320	AreaShape_Zernike_9_7_2	0.016499057	0.016499057
321	Neighbors_AngleBetweenNeighbors_Expanded_0	0.016105948	0.016105948
322	AreaShape_MaxFeretDiameter_0	0.01608521	0.01608521
323	AreaShape_Perimeter_1	0.016032958	0.016032958
324	AreaShape_Zernike_5_1_0	0.015919989	0.015919989
325	Intensity_MeanIntensity_CorrFN_1	0.015544902	0.015544902
326	RadialDistribution_MeanFrac_CorrFN_4of4_1	0.015426987	0.015426987
327	RadialDistribution_MeanFrac_CorrFN_1of4_2	0.015340151	0.015340151
328	AreaShape_Zernike_8_8_2	0.015157205	0.015157205
329	AreaShape_Zernike_8_8_1	0.014763226	0.014763226

330	AreaShape_Zernike_8_4_0	0.013738365	0.013738365
331	Texture_Variance_CorrFN_3_0_2	0.013702567	0.013702567
332	AreaShape_Zernike_6_2_0	0.012850227	0.012850227
333	Intensity_MedianIntensity_CorrFN_2	0.012813002	0.012813002
334	AreaShape_Zernike_7_7_2	0.012699105	0.012699105
335	Texture_InfoMeas1_CorrDAPI_3_0_2	0.011800774	0.011800774
336	AreaShape_MajorAxisLength_0	0.011602042	0.011602042
337	Texture_Contrast_CorrACT_3_0_1	0.011502336	0.011502336
338	Intensity_StdIntensity_CorrFN_2	0.011047436	0.011047436
339	RadialDistribution_MeanFrac_CorrFN_2of4_2	0.010714344	0.010714344
340	RadialDistribution_RadialCV_CorrACT_1of4_1	0.010036371	0.010036371
341	Intensity_MedianIntensity_CorrACT_2	0.009943609	0.009943609
342	Intensity_MinIntensity_CorrFN_2	0.009826137	0.009826137
343	Neighbors_FirstClosestDistance_Expanded_0	0.009530806	0.009530806
344	Intensity_StdIntensityEdge_CorrACT_2	0.007640399	0.007640399
345	Texture_DifferenceEntropy_CorrDAPI_3_0_1	0.006739564	0.006739564
346	Texture_Variance_CorrFN_3_0_0	0.006604816	0.006604816
347	AreaShape_Perimeter_0	0.006550028	0.006550028
348	AreaShape_Zernike_4_0_2	0.006035309	0.006035309
349	Texture_InfoMeas2_CorrDAPI_3_0_0	0.005981091	0.005981091
350	Texture_Entropy_CorrFN_3_0_1	0.005723631	0.005723631
351	Texture_Variance_CorrDAPI_3_0_2	0.005649807	0.005649807
352	AreaShape_Area_1	0.005472249	0.005472249
353	AreaShape_Zernike_1_1_1	0.005464563	0.005464563
354	Texture_Contrast_CorrFN_3_0_2	0.00542861	0.00542861
355	RadialDistribution_FracAtD_CorrACT_1of4_1	0.005410851	0.005410851
356	AreaShape_Zernike_2_0_2	0.004553118	0.004553118
357	AreaShape_Orientation_0	0.004505048	0.004505048
358	AreaShape_Zernike_7_1_2	0.004362279	0.004362279
359	AreaShape_Zernike_3_3_1	0.004296689	0.004296689
360	Texture_SumVariance_CorrDAPI_3_0_2	0.004276061	0.004276061

361	Intensity_MADIntensity_CorrFN_2	0.003963061	0.003963061
362	Texture_InfoMeas2_CorrACT_3_0_0	0.003952856	0.003952856
363	Texture_SumAverage_CorrACT_3_0_2	0.003430732	0.003430732
364	AreaShape_Orientation_1	0.002957359	0.002957359
365	Neighbors_PercentTouching_Expanded_0	0.002881397	0.002881397
366	AreaShape_Zernike_3_3_0	0.002735426	0.002735426
367	AreaShape_Zernike_9_5_0	0.002545077	0.002545077
368	Texture_SumVariance_CorrFN_3_0_2	0.002450483	0.002450483
369	Intensity_MedianIntensity_CorrFN_1	0.002275261	0.002275261
370	AreaShape_Zernike_5_3_1	0.001998395	0.001998395
371	AreaShape_Zernike_6_4_0	0.001996358	0.001996358
372	Texture_SumAverage_CorrACT_3_0_0	0.001979678	0.001979678
373	Texture_Entropy_CorrACT_3_0_0	0.001939507	0.001939507
374	Texture_InverseDifferenceMoment_CorrACT_3_0_2	0.001898719	0.001898719
375	Texture_Correlation_CorrACT_3_0_1	0.001844806	0.001844806
376	Texture_SumAverage_CorrACT_3_0_1	0.001603636	0.001603636
377	Texture_SumVariance_CorrACT_3_0_2	0.001573037	0.001573037
378	Texture_DifferenceVariance_CorrFN_3_0_1	0.001566701	0.001566701
379	AreaShape_Zernike_4_2_1	0.001474742	0.001474742
380	Texture_Entropy_CorrDAPI_3_0_1	0.001312661	0.001312661
381	RadialDistribution_RadialCV_CorrFN_3of4_1	0.001294841	0.001294841
382	AreaShape_Solidity_0	0.001198277	0.001198277
383	Intensity_UpperQuartileIntensity_CorrACT_2	0.00095392	0.00095392
384	AreaShape_Area_0	0.000925862	0.000925862
385	AreaShape_Zernike_9_3_1	0.000916562	0.000916562
386	AreaShape_Zernike_4_0_1	0.00082497	0.00082497
387	Texture_DifferenceEntropy_CorrFN_3_0_2	0.000701506	0.000701506
388	Texture_DifferenceEntropy_CorrDAPI_3_0_2	0.000551724	0.000551724
389	AreaShape_Zernike_8_6_0	0.000538798	0.000538798
390	AreaShape_Zernike_9_5_1	0.000534267	0.000534267
391	Texture_Gabor_CorrFN_3_1	0.000510658	0.000510658

392	AreaShape_MinorAxisLength_2	0.000452481	0.000452481
393	AreaShape_MaximumRadius_1	0.000415525	0.000415525
394	RadialDistribution_MeanFrac_CorrFN_1of4_1	0.000324058	0.000324058
395	AreaShape_MinFeretDiameter_2	0.000305264	0.000305264
396	RadialDistribution_FracAtD_CorrFN_1of4_1	0.000235107	0.000235107
397	RadialDistribution_MeanFrac_CorrFN_4of4_2	0.000196443	0.000196443
398	AreaShape_MeanRadius_0	0.000159291	0.000159291
399	Texture_Correlation_CorrDAPI_3_0_1	8.94761E–05	8.94761E–05
400	AreaShape_Zernike_3_1_1	8.19065E–05	8.19065E–05
401	Texture_SumEntropy_CorrACT_3_0_2	7.74509E–05	7.74509E–05
402	AreaShape_Zernike_5_1_1	6.29617E–05	6.29617E–05
403	Texture_Correlation_CorrACT_3_0_2	6.12988E–05	6.12988E–05
404	AreaShape_EulerNumber_1	4.63145E–05	4.63145E–05
405	RadialDistribution_FracAtD_CorrFN_4of4_1	3.9456E–05	3.9456E–05
406	AreaShape_Zernike_8_4_1	2.26027E–05	2.26027E–05
407	RadialDistribution_MeanFrac_CorrACT_1of4_2	2.02807E–05	2.02807E–05
408	Texture_Contrast_CorrACT_3_0_2	5.27896E–06	5.27896E–06
409	AreaShape_Zernike_6_4_1	1.78849E–06	1.78849E–06

S1 Table Legend: The features names can be read as follows: The first name, e.g. Granularity, specifies the computed feature with a certain parameter after the underscore. The second term specifies the channel name on which the feature has been calculated e.g. α –SMA / Actin or FN. FN stands for Fibronectin, ACT for α–SMA / Actin. The ‘Corr’ indicates that the channel image was corrected using a background correction algorithm. E.g. the feature "Granularity_1_CorrFN_2" detects the object distribution of small speckles with a radius of one pixel in the FN channel. Details on feature description can be found here: http: / cellprofiler–manual.s3.amazonaws.com / CellProfiler–3.0.0 / modules / measurement.html#
