# Supplementary material for: Novel high–throughput myofibroblast assays identify agonists with therapeutic potential in pulmonary fibrosis that act via EP2 and EP4 receptors
Source: PLoS One. 2018 Nov 28;13(11):e0207872. doi: 10.1371/journal.pone.0207872 (PMC6261607; doi:10.1371/journal.pone.0207872)
Supplement: S2 Table — (PDF) [file pone.0207872.s002.pdf]

S2 Table. The 42 hits identified by screening 1,585 approved drugs with the HCA.

Name	Effect HCA at 10 µM [% inhibition]	Effect on viability at 10 µM [% reduction]	IC50 HCA [nM]	IC50 nuclear count [nM]
Camptothecine (S+)	105	5	41	>25,000
Proscillaridin A	101	12	1	>25,000
Carfilzomib (PR–171)	100	52	4	20,100
Ouabain	100	15	12	>25,000
Lanatoside C	100	19	35	>25,000
Emetine	100	25	71	>25,000
Digoxigenin	100	11	220	>25,000
Alprostadil	97	–19	9	>25,000
Digitoxigenin	93	22	39	>25,000
Digoxin	87	19	10	>25,000
Daunorubicin hydrochloride	73	14	270	20,200
Cycloheximide	91	27	109	>25,000
Mitoxantrone dihydrochloride	101	31	620	19,000
Doxorubicin hydrochloride	64	20	450	>25,000
Topotecan	88	8	970	>25,000
Dasatinib (BMS–354825)	89	–10	1,020	>25,000
Epirubicin hydrochloride	93	28	1,070	18,000
Haloprogin	77	61	1,300	4,500
Crizotinib (PF–02341066)	63	66	1,500	16,000
Thiostrepton	85	–11	2,200	7,600
Dabrafenib (GSK2118436)	56	9	2,900	>25,000
Azacytidine–5	97	–4	3,200	>25,000
Etravirine (TMC125)	100	29	3,900	>25,000
Nifedipine	79	29	4,700	7,300
Vorinostat	101	24	5,400	>25,000
Benzethonium chloride	78	72	5,500	13,000
Chlorhexidine	80	24	5,800	12,000
Dequalinium dichloride	67	39	5,800	>25,000
Bazedoxifene HCl	89	55	5,800	9,070
Chloroxine	80	14	5,900	>25,000
Aminacrine	97	2	6,000	24,000
Halofantrine hydrochloride	101	29	6,020	5,200
Hexachlorophene	93	8	6,700	>25,000
Fenbendazole	76	51	6,700	5,800

Astemizole	105	49	6,800	8,300
Ethacridine lactate monohydrate	73	–9	7,500	>25,000
PCI–32765 (Ibrutinib)	67	22	7,600	>25,000
Cilnidipine	92	15	8,040	>25,000
Indatraline hydrochloride	67	20	8,060	11,000
Thiethylperazine dimalate	76	34	8,500	13,000
Estradiol Valerate	66	65	9,700	11,000
GBR 12909 dihydrochloride	56	13	9,900	12,000
