# Supplementary material for: Novel high–throughput myofibroblast assays identify agonists with therapeutic potential in pulmonary fibrosis that act via EP2 and EP4 receptors
Source: PLoS One. 2018 Nov 28;13(11):e0207872. doi: 10.1371/journal.pone.0207872 (PMC6261607; doi:10.1371/journal.pone.0207872)
Supplement: S3 Table — (PDF) [file pone.0207872.s003.pdf]

S3 Table. Selectivity and potency of EP_2 receptor agonists.

Receptor / Readout	EP2 / cAMP		EP4 / cAMP		EP2 / β–arrestin		EP4 / β–arrestin	
**Name**	**EC50 [nM]**	**Emax (%)**	**EC50 [nM]**	**Emax (%)**	**EC50 [nM]**	**Emax (%)**	**EC50 [nM]**	**Emax (%)**
Alprostadil	0.04	100	10	110	520	104	9	110
PGE2	0.04	100	3	110	110	103	4	107
ONO–18 c	<0.01	98	5	64	52	160	5,000	26
ONO–18 k	0.03	99	>10,000	24	110	32	>10,000	15
Evatanepag	0.6	100	>10,000	32	290	41	>10,000	10

n.a.: not assessed.
