# Supplementary material for: Novel high–throughput myofibroblast assays identify agonists with therapeutic potential in pulmonary fibrosis that act via EP2 and EP4 receptors
Source: PLoS One. 2018 Nov 28;13(11):e0207872. doi: 10.1371/journal.pone.0207872 (PMC6261607; doi:10.1371/journal.pone.0207872)
Supplement: S4 Table — (PDF) [file pone.0207872.s004.pdf]

S4 Table. Effect of TGF-β1 on the α-SMA / tubulin and COL1 / tubulin ratios in NHLF and RLMyoF

	**NHLF**		**RLMyoF**	
	**Control**	**TGF-β1[1]**	**Control**	**TGF-β1**
α-SMA / tubulin	0.12 (n = 1)	0.51 ± 0.06 (n = 6)	1.60 ± 0.15 (n = 5)	1.67 (n = 1)
COL1 / tubulin	0.05 (n = 1)	0.33 ± 0.07 (n = 6)	0.29 ± 0.04 (n = 5)	0.36 (n = 1)

[1]5ng/mL TGF-β1

5
