# Supplementary figures and images for: Novel high–throughput myofibroblast assays identify agonists with therapeutic potential in pulmonary fibrosis that act via EP2 and EP4 receptors
Source: PLoS One. 2018 Nov 28;13(11):e0207872. doi: 10.1371/journal.pone.0207872 (PMC6261607; doi:10.1371/journal.pone.0207872)

S1 Fig

A

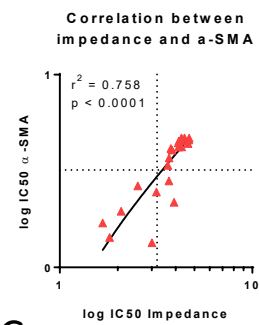

B

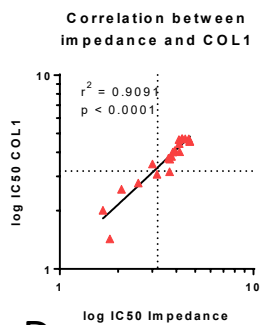

C

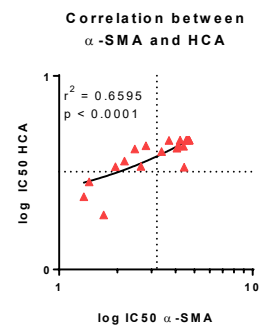

D

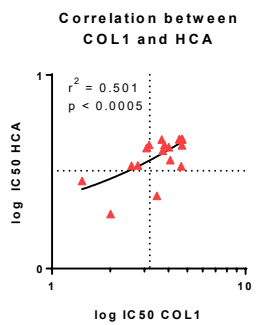

E

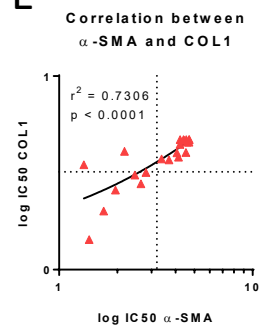

F

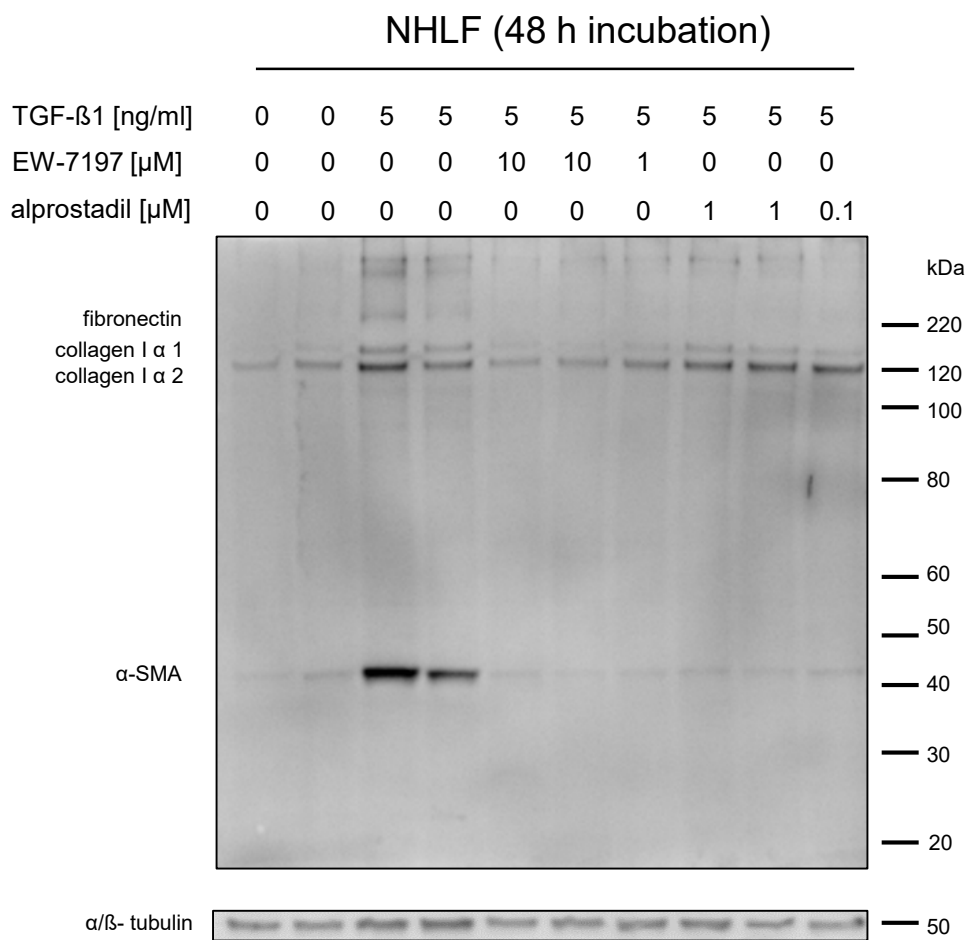

Supplement: S1 Fig — IC50 data was log transformed using Graph Pad Prism and linear regression was performed on the logarithms. (A) Plot of impedance and α–SMA data. (B) Plot of impedance and COL1 data. (C) Plot of HCA and α–SMA data. (D) Plot of HCA and COL1 data. (E) Plot of α–SMA and COL1 data. (F) Primary NHLF were serum starved for 24 h and stimulated either with 5 ng / ml TGF–β1 (TGF–β) or with the appropriate vehicle control, in presence or absence of the ALK5 blocker EW-7197 or alprostadil for 48 h. α–SMA (42 kDa, Sigma # A2547), α / β–tubulin (50 kDa, CellSignaling # 2148), collagen 1 α 1 (139 kDa, Aviva Systems Biology # OAMA03716) and fibronectin (~250 kDa, Santa Cruz Biotech # sc-6952) were visualized by immunoblot analysis. The protein molecular weight marker (Invitrogen # LC5925) was run in parallel to estimate protein size. IPQA data were generated from NHLF donor 1 with 5 ng / ml TGF–β1 in presence compound and / or 0.5% DMSO (vehicle) for 48 h; n = 1. HCA data were generated from NHLF donor 2 treated with 5 ng / ml TGF–β1 in presence compound and / or 0.5% DMSO (vehicle) for 48 h; mean of n = 2. R square (r2) and p value of linear regression are indicated. P < 0.05 was considered significant. (PDF) [file pone.0207872.s005.pdf]

S2 Fig

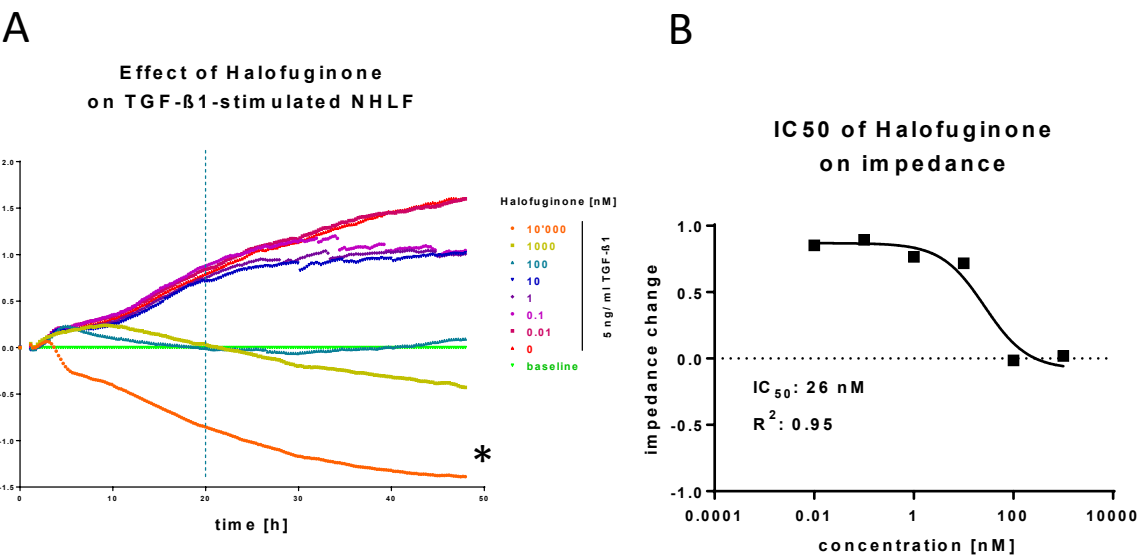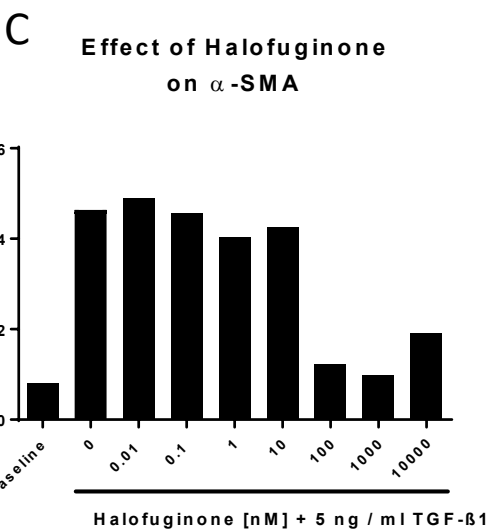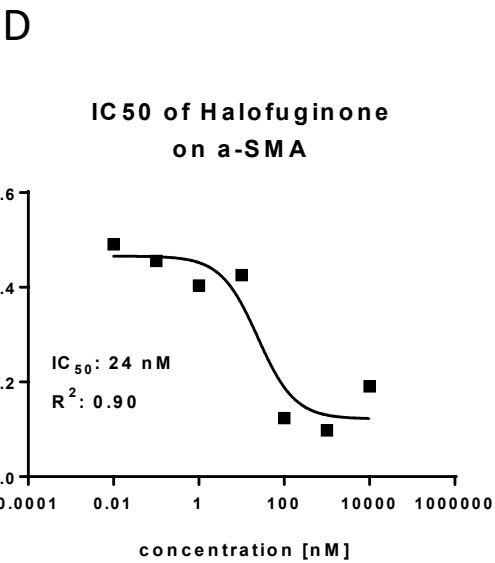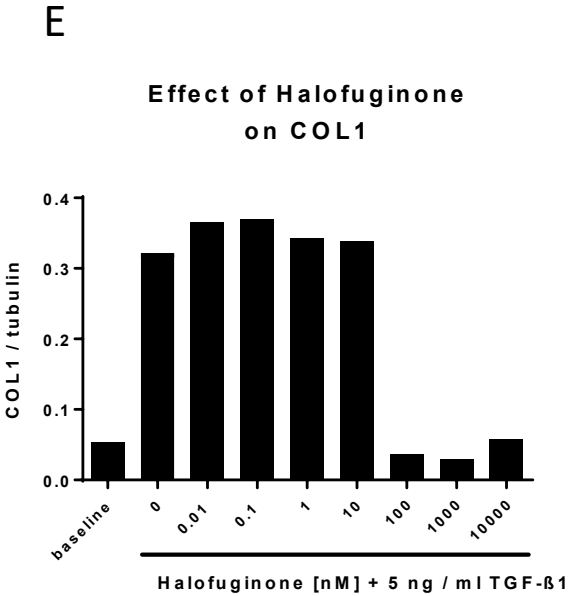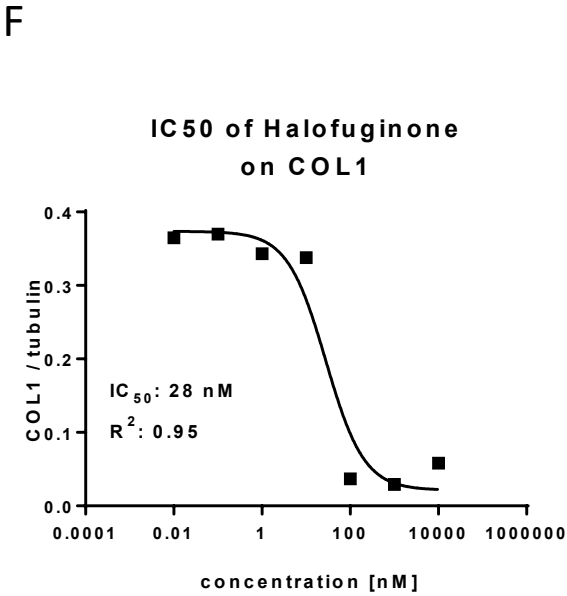

Supplement: S2 Fig — Shown are impedance traces of non–stimulated NHLF cells (0 ng / ml TGF–β1; baseline, green), NHLF cells stimulated with 5ng / ml TGF–β1 in the absence of compound (0 nM compound, red), and NHLF cells exposed to dilutions series of halofuginone (0.01–10,000 nM). Due to cytotoxicity the impedance data corresponding to 10,000 nM halofuginone (marked with an asterisk) were excluded for the IC50 calculation (A). Concentration response curves of halofuginone in presence of 5 ng / ml TGF–β1 where then generated with baseline (0 ng / ml TGF–β1) subtracted impedance values at t = 20 h post TGF–β1 addition (B). At t = 48 h after TGF–β1 addition the cells were lysed and α–SMA (C) and COL1 (E) were quantified by MS / MS. Bars represent protein data normalized to tubulin. Concentration response curves of halofuginone in presence of 5 ng / ml TGF–β1 where then generated with the normalized α–SMA (D) and COL1 (F) data. One of two very similar experiments is shown. (PDF) [file pone.0207872.s006.pdf]

# S4 Fig

A

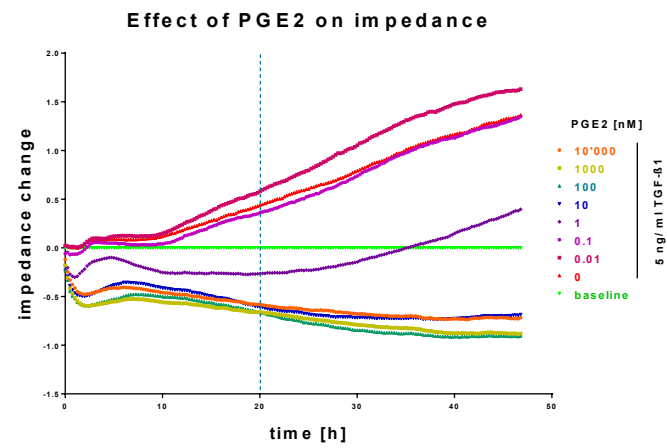

B

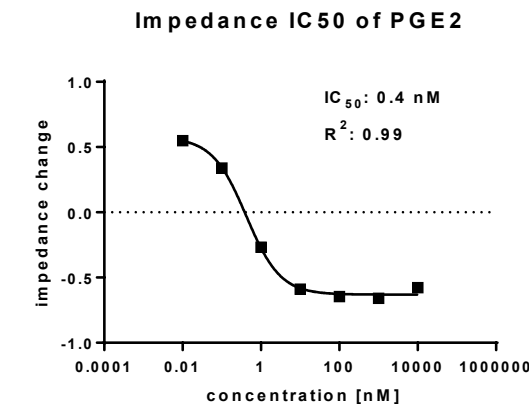

C

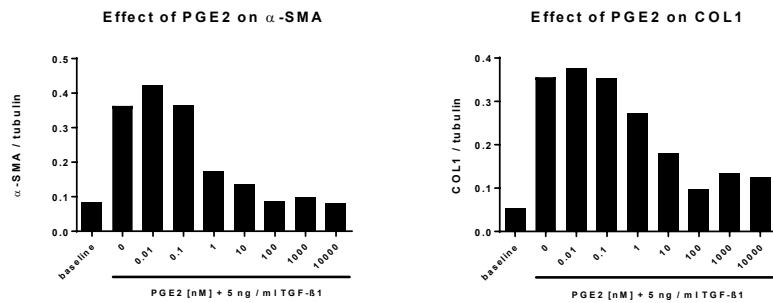

D

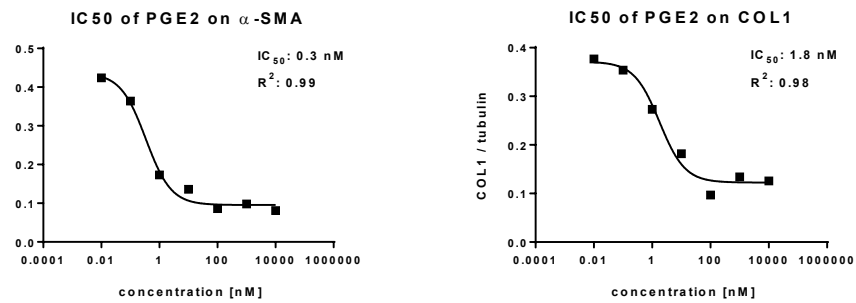

Supplement: S4 Fig — Impedance recordings of non–stimulated NHLF cells (0 ng / ml TGF–β1; baseline, green), NHLF cells stimulated with 5ng / ml TGF–β1 in the absence of compound (0 nM compound, red), and NHLF cells exposed to dilutions series of PGE2 (0.01–10,000 nM) (A). Concentration response curves of PGE2 in presence of 5 ng / ml TGF–β1 where generated with baseline (0 ng / ml TGF–β1) subtracted impedance values at t = 20 h post TGF–β1 addition (B). At t = 48 h after TGF–β1 addition the cells were lysed and α–SMA and COL1 were quantified by MS / MS. Bars represent protein data obtained from a single well normalized to tubulin from NHLF cells incubated with dilution series of PGE2 followed by stimulation with 5 ng / ml TGF–β1 (C). Concentration response curves in presence of 5 ng / ml TGF–β1 where generated with the normalized α–SMA and COL1 data (D). (PDF) [file pone.0207872.s008.pdf]

S5 Fig  
A

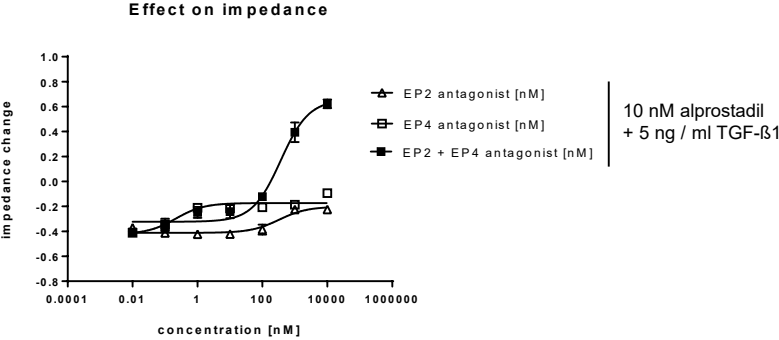

B

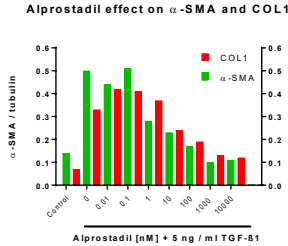

C

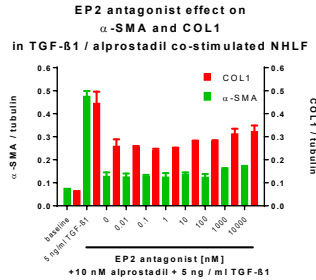

D

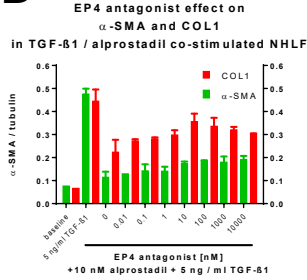

E

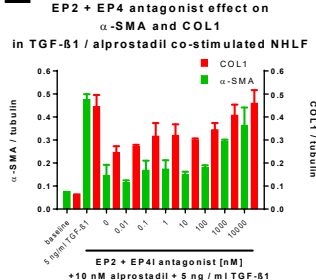

Supplement: S5 Fig — (A) Impedance changes of NHLF cells incubated with 10 nM alprostadil and 5 ng / ml TGF–β1, exposed to dilution series of an EP2 receptor antagonist, and EP4 receptor antagonist or the combination of the EP2 and the EP4 antagonist, were exported at t = 20 h and plotted against the compound concentration to generate concentration response curves. (B) At t = 0 h NHLF fibroblasts were stimulated with 5 ng / ml TGF–β1 and incubated with dilution series (0.01–10,000 nM) of alprostadil (B), or the EP2 prostaglandin receptor antagonist (C), the EP4 receptor antagonist (D), and of both the EP2 and the EP4 receptor antagonists (E) in the absence (B) or in presence (C–E) of 10 nM alprostadil. At t = 48 h after TGF–β1 addition the cells were lysed and α–SMA and COL1 were quantified by MS / MS. Bars represent protein data normalized to tubulin. Data of one representative experiment is shown in B. Data shown in C–E represent mean ± SD (n = 2). (PDF) [file pone.0207872.s009.pdf]

S8 Fig

A

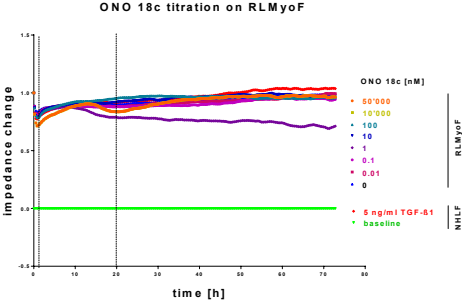

B

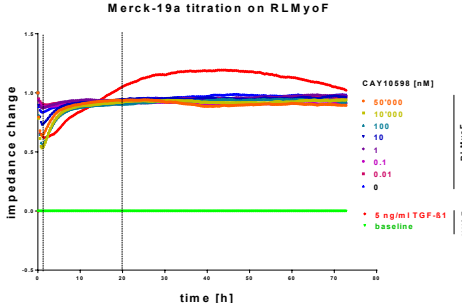

C

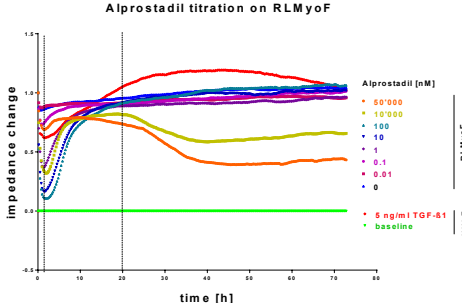

D

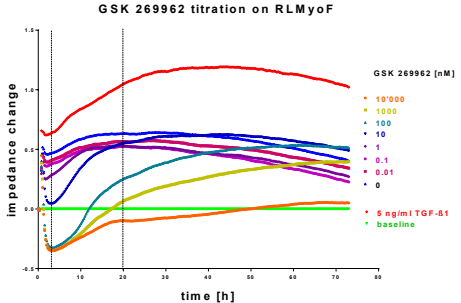

E

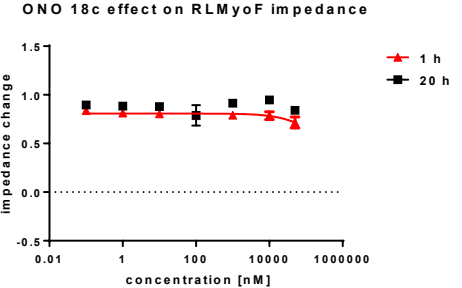

F

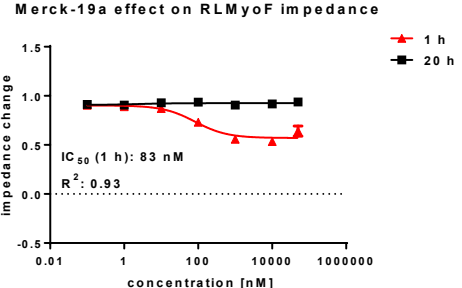

G

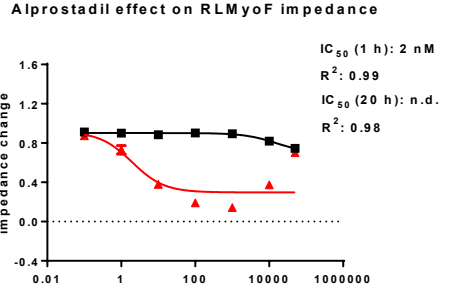

H

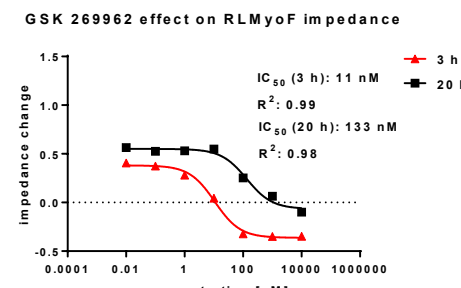

Supplement: S8 Fig — (A-C) Impedance recordings of non–stimulated RLMyoF that were starved for 24 h and then exposed for further 72 h to dilution series (0.01–10,000 nM) of the selective EP2 receptor agonists ONO–18c (A, D), alprostadil (B, E), or the ROCK1/2 inhibitor GSK-269962 (C, F) are shown. Impedance traces of non–stimulated vehicle–treated NHLF cells (0 ng / ml TGF–β1; baseline) and of NHLF fibroblasts stimulated with 5ng / ml TGF–β1 (0 nM agonist; vehicle) are shown for comparison in green and red color, respectively. (D-F) Impedance changes at t = 3 h and t = 20 h were exported and plotted against the agonist concentration for IC50 calculation. (PDF) [file pone.0207872.s012.pdf]
