# Supplementary material for: Novel high–throughput myofibroblast assays identify agonists with therapeutic potential in pulmonary fibrosis that act via EP2 and EP4 receptors
Source: PLoS One. 2018 Nov 28;13(11):e0207872. doi: 10.1371/journal.pone.0207872 (PMC6261607; doi:10.1371/journal.pone.0207872)
Supplement: S3 Fig — The effect of TGF–β1 (5 ng / ml) is inhibited by increasing concentrations (0.016 nM– 25’000 nM) of the cardiac glycoside digoxigenin as captured by high–content confocal microscopy 48 h after TGF–β1 stimulation. Nuclei are stained with DAPI, α–SMA, FN and the cytosol with anti–α–SMA IgG, anti–FN IgG and CellMaskTM Orange, respectively (A). Impedance recordings of non–stimulated NHLF cells (0 ng / ml TGF–β1; baseline, green), NHLF cells stimulated with 5ng / ml TGF–β1 in the absence of compound (0 nM compound, red), and NHLF cells exposed to dilutions series of digoxigenin (0.004–4,000 nM) (B). Concentration response curves of digoxigenin in presence of 5 ng / ml TGF–β1 where then generated with baseline (0 ng / ml TGF–β1) subtracted impedance values at t = 20 h post TGF–β1 addition. One of two very similar experiments is shown. (C). At t = 48 h after TGF–β1 addition the cells were lysed and α–SMA and COL1 were quantified by MS / MS. Bars represent mean (n = 2) of protein amount normalized to tubulin (D). (PDF) [file pone.0207872.s007.pdf]

S3 Fig

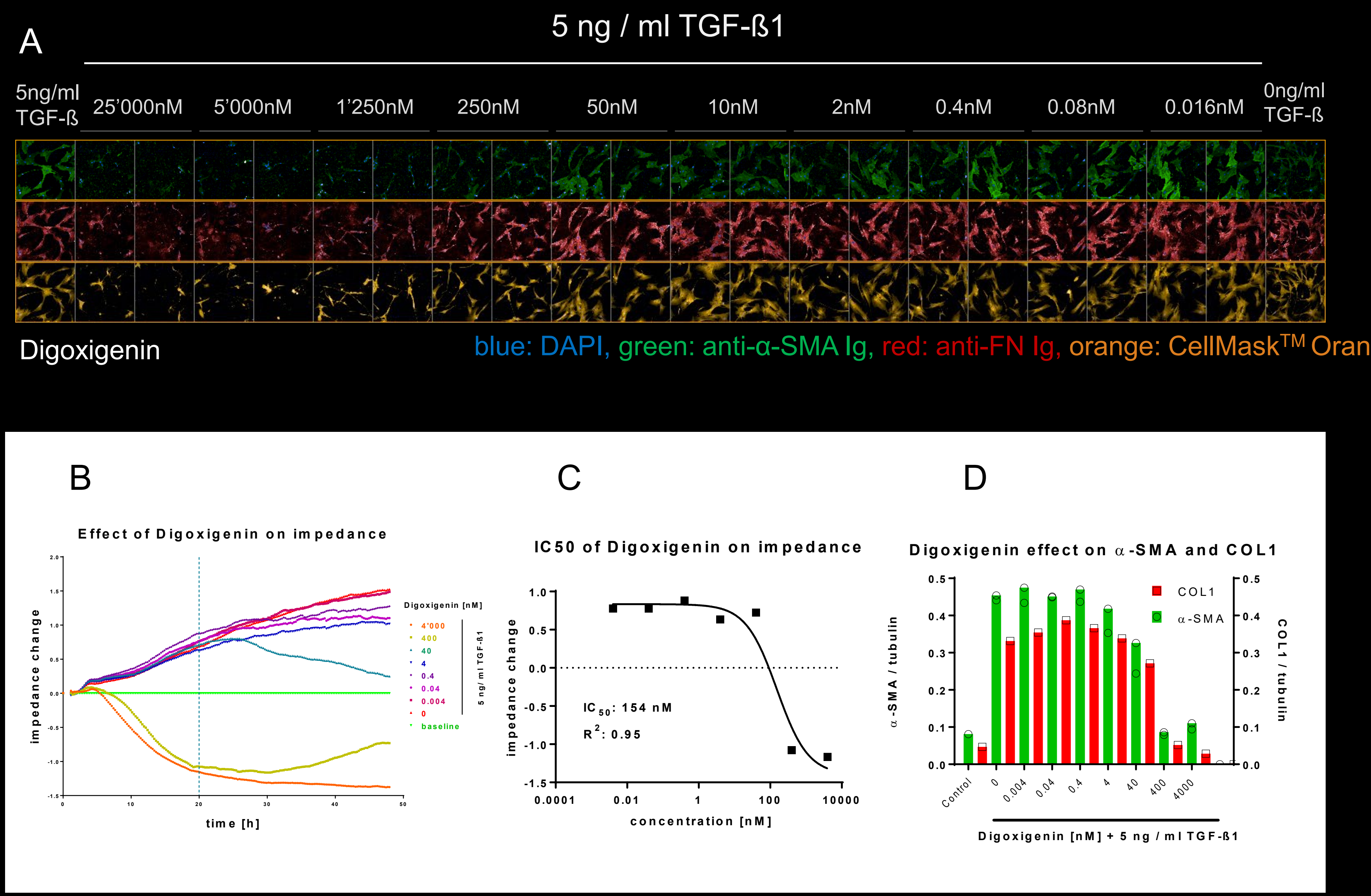
A
5 ng / ml TGF-ß1
5ng/ml TGF-ß
25’000nM
5’000nM
1’250nM
250nM
50nM
10nM
2nM
0.4nM
0.08nM
0.016nM
0ng/ml TGF-ß
Digoxigenin
blue: DAPI, green: anti-α-SMA Ig, red: anti-FN Ig, orange: CellMask™ Oran
B
Effect of Digoxigenin on impedance
impedance change
time [h]
Digoxigenin [nM]
4'000
400
40
4
0.4
0.04
0.004
0
baseline
5 ng/ ml TGF-ß1
C
IC50 of Digoxigenin on impedance
impedance change
concentration [nM]
IC50: 154 nM
R2: 0.95
D
Digoxigenin effect on α-SMA and COL1
α-SMA / tubulin
COL1 / tubulin
COL1
α-SMA
Control
Digoxigenin [nM] + 5 ng / ml TGF-ß1
