# Supplementary material for: Novel high–throughput myofibroblast assays identify agonists with therapeutic potential in pulmonary fibrosis that act via EP2 and EP4 receptors
Source: PLoS One. 2018 Nov 28;13(11):e0207872. doi: 10.1371/journal.pone.0207872 (PMC6261607; doi:10.1371/journal.pone.0207872)
Supplement: S6 Fig — (A) Impedance recordings of NHLF cells exposed to dilution series of the selective EP2 receptor agonists ONO–18c, ONO–18k, and evatanepag (0.01–10,000 nM), followed by stimulation with TGF–β1 (5ng / ml). Impedance traces of non–stimulated vehicle–treated NHLF cells (0 ng / ml TGF–β1; baseline) and of NHLF fibroblasts stimulated with 5ng / ml TGF–β1 (0 nM agonist; vehicle) are highlighted in green and red color, respectively. Impedance changes at t = 20 h were exported and plotted against the agonist concentration for IC50 calculation. (B) At t = 48 h after TGF–β1 addition the cells were lysed and α–SMA and COL1 were quantified by MS / MS. Bars represent protein data normalized to tubulin. (C) Concentration response curves were generated for alprostadil and the selective EP2 agonists ONO–18c, ONO–18k, and evatanepag of normalized α–SMA (top row) and COL1 (bottom row) from NHLF fibroblasts stimulated with 5 ng / ml TGF–β1. (PDF) [file pone.0207872.s010.pdf]

S6 Fig

A

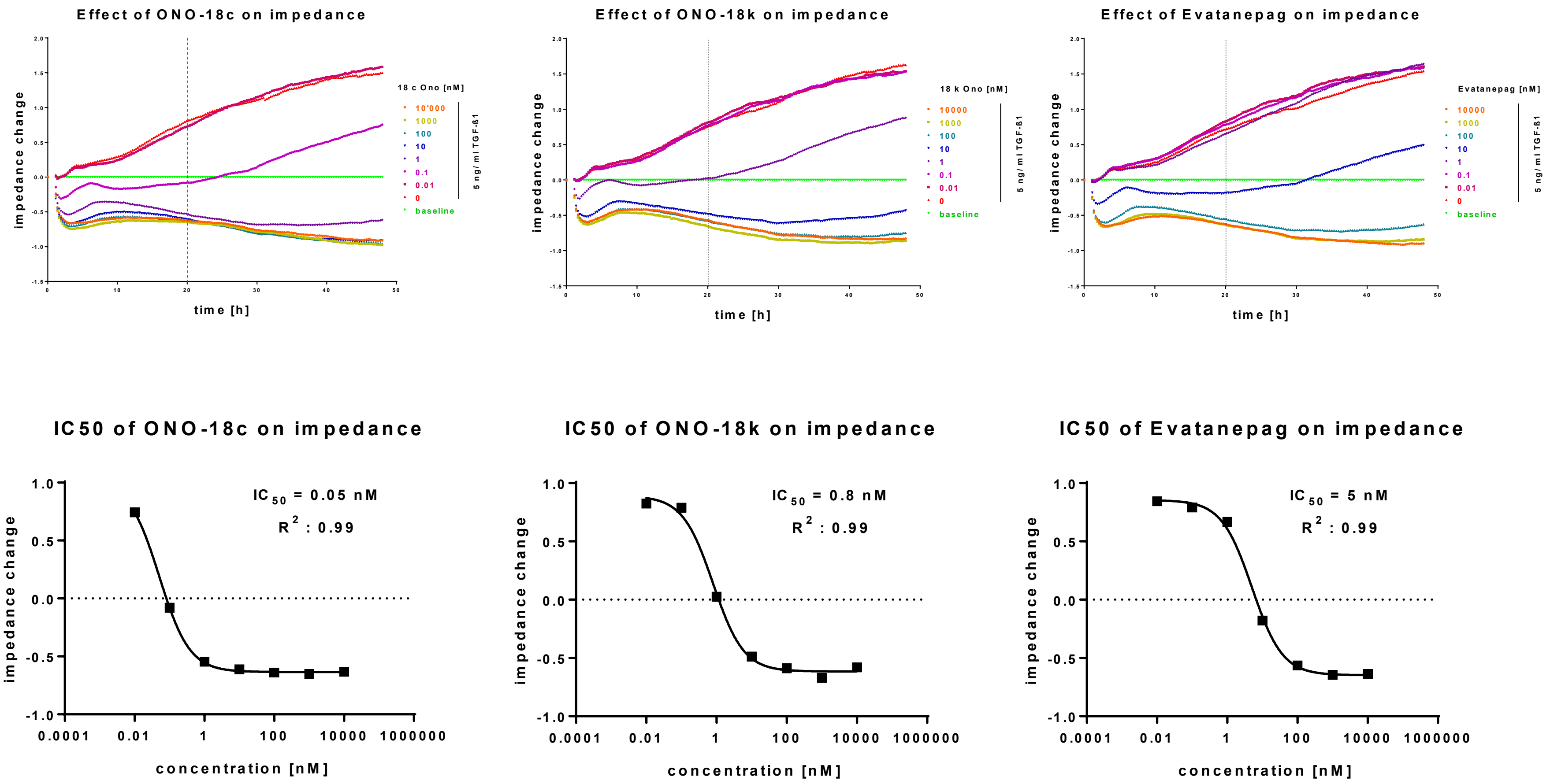
Effect of ONO-18c on impedance
impedance change
time [h]
18 c Ono [nM]
10'000
1000
100
10
1
0.1
0.01
0
baseline
5 ng/ml TGF-ß1
Effect of ONO-18k on impedance
impedance change
time [h]
18 k Ono [nM]
10000
1000
100
10
1
0.1
0.01
0
baseline
5 ng/ml TGF-ß1
Effect of Evatanepag on impedance
impedance change
time [h]
Evatanepag [nM]
10000
1000
100
10
1
0.1
0.01
0
baseline
5 ng/ml TGF-ß1
IC50 of ONO-18c on impedance
IC50 = 0.05 nM
R2 : 0.99
impedance change
concentration [nM]
IC50 of ONO-18k on impedance
IC50 = 0.8 nM
R2 : 0.99
impedance change
concentration [nM]
IC50 of Evatanepag on impedance
IC50 = 5 nM
R2 : 0.99
impedance change
concentration [nM]

B

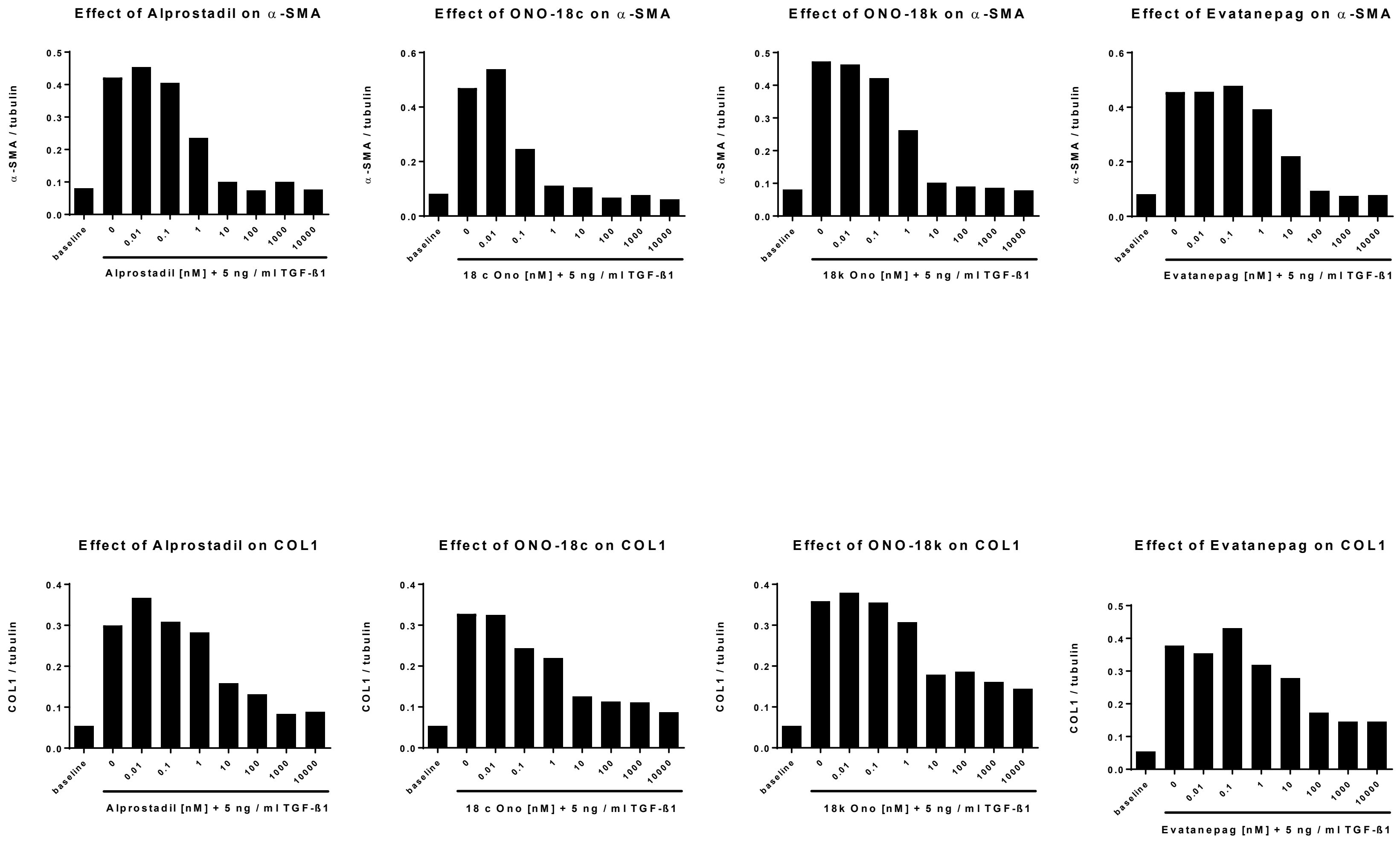

Effect of Alprostadil on α-SMA
α-SMA / tubulin
0.0
0.1
0.2
0.3
0.4
0.5
baseline
0
0.01
0.1
1
10
100
1000
10000
Alprostadil [nM] + 5 ng / ml TGF-ß1
Effect of ONO-18c on α-SMA
α-SMA / tubulin
0.0
0.2
0.4
0.6
18 c Ono [nM] + 5 ng / ml TGF-ß1
Effect of ONO-18k on α-SMA
α-SMA / tubulin
18k Ono [nM] + 5 ng / ml TGF-ß1
Effect of Evatanepag on α-SMA
α-SMA / tubulin
Evatanepag [nM] + 5 ng / ml TGF-ß1
Effect of Alprostadil on COL1
COL1 / tubulin
Alprostadil [nM] + 5 ng / ml TGF-ß1
Effect of ONO-18c on COL1
COL1 / tubulin
18 c Ono [nM] + 5 ng / ml TGF-ß1
Effect of ONO-18k on COL1
COL1 / tubulin
18k Ono [nM] + 5 ng / ml TGF-ß1
Effect of Evatanepag on COL1
COL1 / tubulin
Evatanepag [nM] + 5 ng / ml TGF-ß1

C

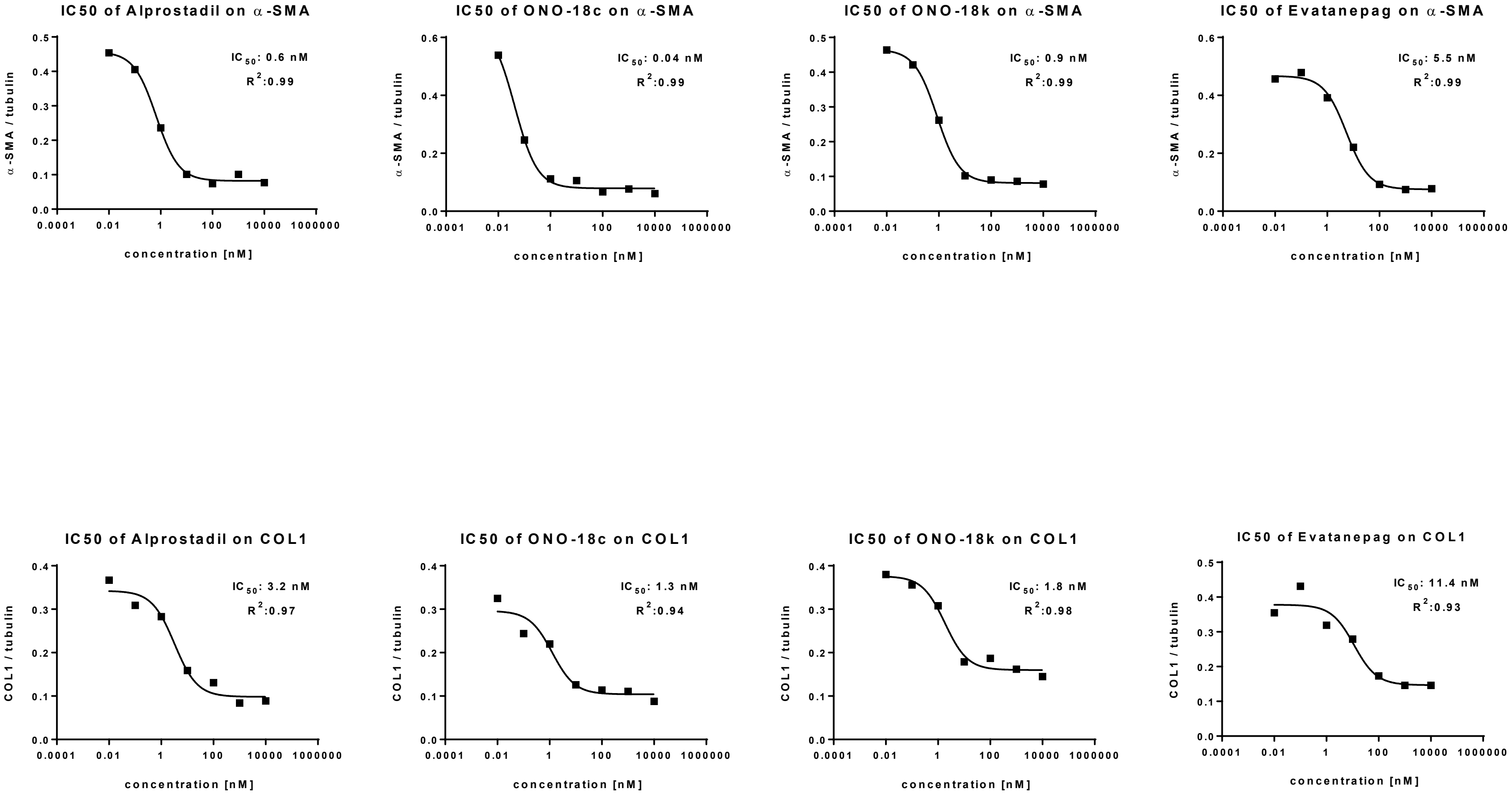

IC50 of Alprostadil on α-SMA
IC50: 0.6 nM
R2:0.99
IC50 of ONO-18c on α-SMA
IC50: 0.04 nM
R2:0.99
IC50 of ONO-18k on α-SMA
IC50: 0.9 nM
R2:0.99
IC50 of Evatanepag on α-SMA
IC50: 5.5 nM
R2:0.99
α-SMA / tubulin
IC50 of Alprostadil on COL1
IC50: 3.2 nM
R2:0.97
IC50 of ONO-18c on COL1
IC50: 1.3 nM
R2:0.94
IC50 of ONO-18k on COL1
IC50: 1.8 nM
R2:0.98
IC50 of Evatanepag on COL1
IC50: 11.4 nM
R2:0.93
COL1 / tubulin
0.0001
0.01
1
100
10000
1000000
concentration [nM]
