# Supplementary material for: Novel high–throughput myofibroblast assays identify agonists with therapeutic potential in pulmonary fibrosis that act via EP2 and EP4 receptors
Source: PLoS One. 2018 Nov 28;13(11):e0207872. doi: 10.1371/journal.pone.0207872 (PMC6261607; doi:10.1371/journal.pone.0207872)
Supplement: S7 Fig — NHLF cells were differentiated for 24 h with 5 ng / ml TGF–β1 into myofibroblasts. The cells were then washed 3 times to remove TGF–β1 and incubated with increasing concentrations (0–25,000 nM) of the cardiac glycosides digoxigenin, digoxin and digitoxigenin (A), with halofuginione or with nintedanib / BIBF1120 (B) for 72 h in starvation medium. Cells were fixed and immunostained to detect the myofibroblast markers FN and α–SMA (green) by high–content confocal microscopy. (C) Impedance recordings of NHLF cells pre–stimulated for 24 h with 5 ng / ml TGF–β1 treated with dilution series (0.01–10,000 nM) of the ALK5 blocker EW–7197, alprostadil, PGE2, of the selective EP2 agonists ONO–18c, ONO–18k and evatanepag, of the adenylyl cyclase activator forskolin, as well as of the antifibrotic halofuginone and the cardiac glycoside digoxigenin. Impedance traces of non–stimulated NHLF cells (0 ng / ml TGF–β1; baseline) and of NHLF fibroblasts stimulated with 5ng / ml TGF–β1 (0 nM agonist; vehicle) are highlighted in green and red color, respectively. IC50 concentrations were calculated from normalized MS / MS data to quantify the effect of increasing concentrations (0.01–10,000 nM) of EW–7197, alprostadil, PGE2, ONO–18c, ONO–18k, evatanepag, forskolin, halofuginone, as well as digoxigenin, on accumulated α–SMA (D) and COL1 (E) in NHLF fibroblasts pre–stimulated with 5 ng / ml TGF–β1 for 24h. Representative experiments are shown. (PDF) [file pone.0207872.s011.pdf]

S7 Fig

A

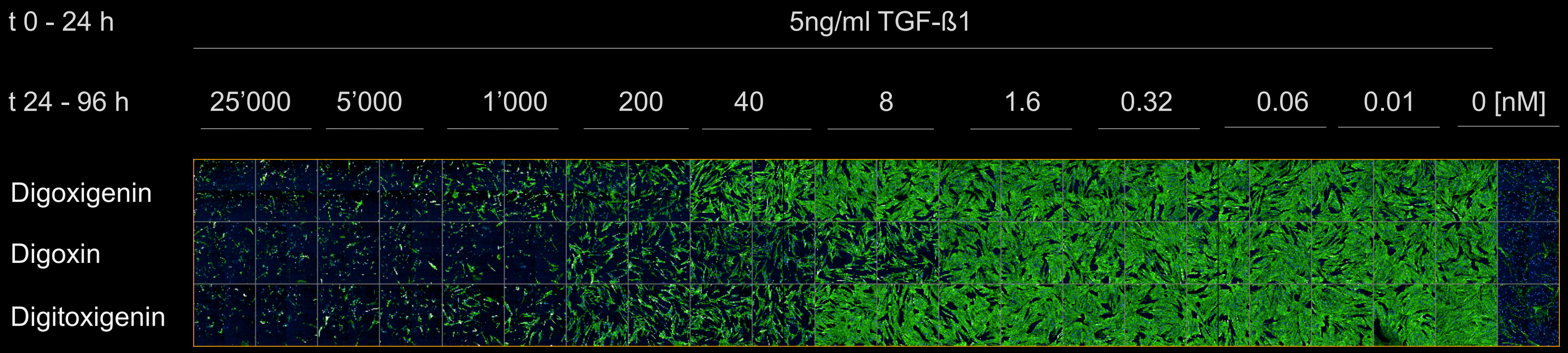

t 0 - 24 h
5ng/ml TGF-ß1
t 24 - 96 h
25'000
5'000
1'000
200
40
8
1.6
0.32
0.06
0.01
0 [nM]
Digoxigenin
Digoxin
Digitoxigenin

B

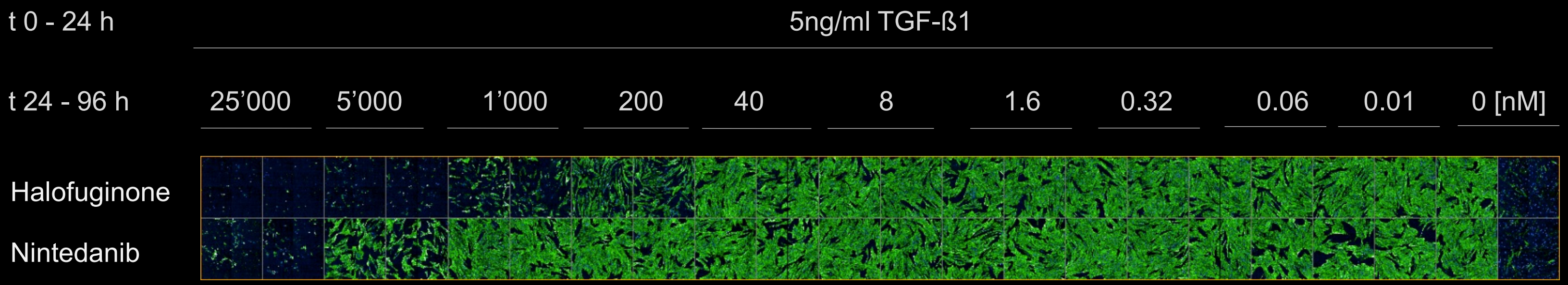

t 0 - 24 h
5ng/ml TGF-ß1
t 24 - 96 h
25'000
5'000
1'000
200
40
8
1.6
0.32
0.06
0.01
0 [nM]
Halofuginone
Nintedanib

C

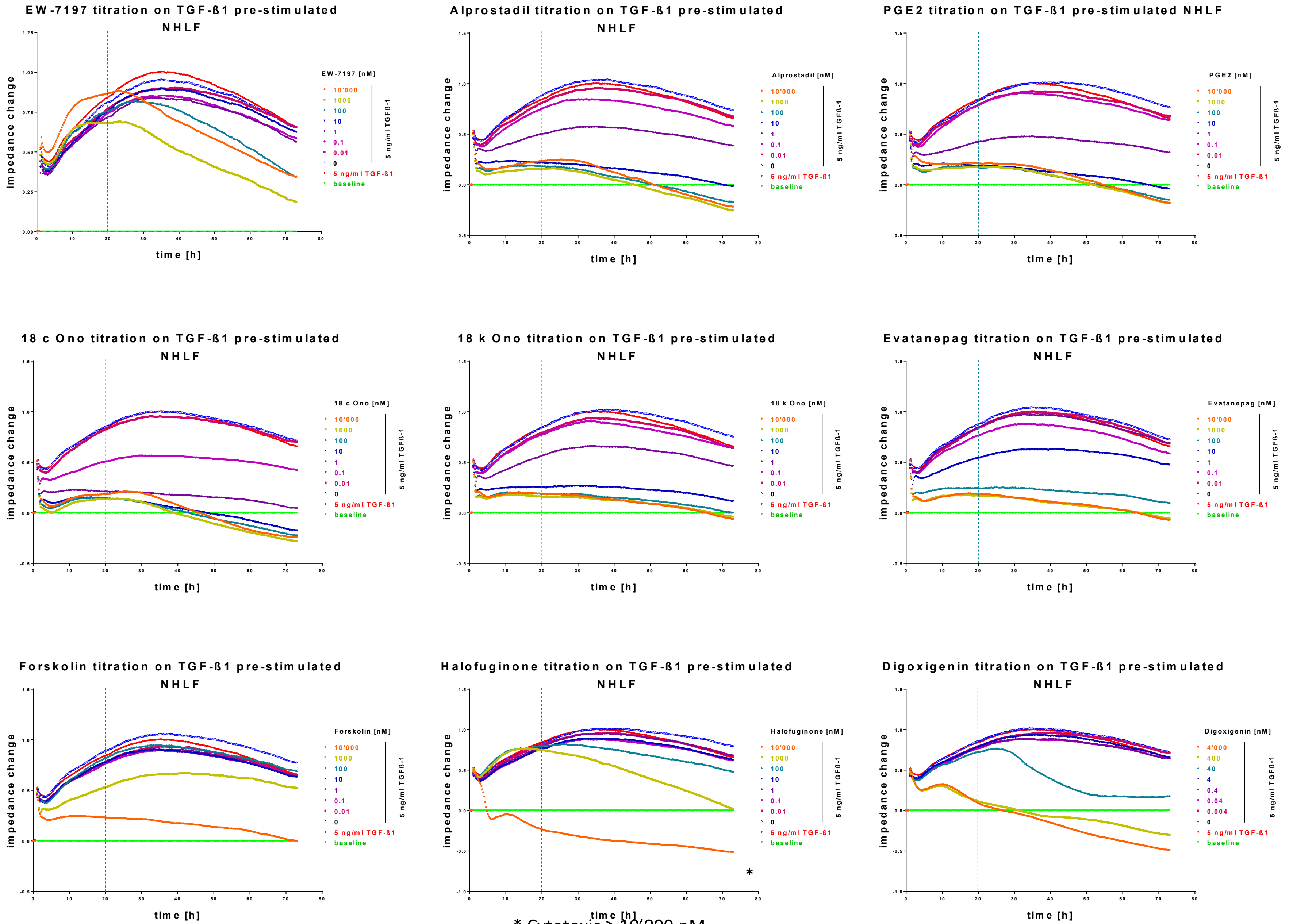

* Cytotoxic ≥ 10’000 nM

D

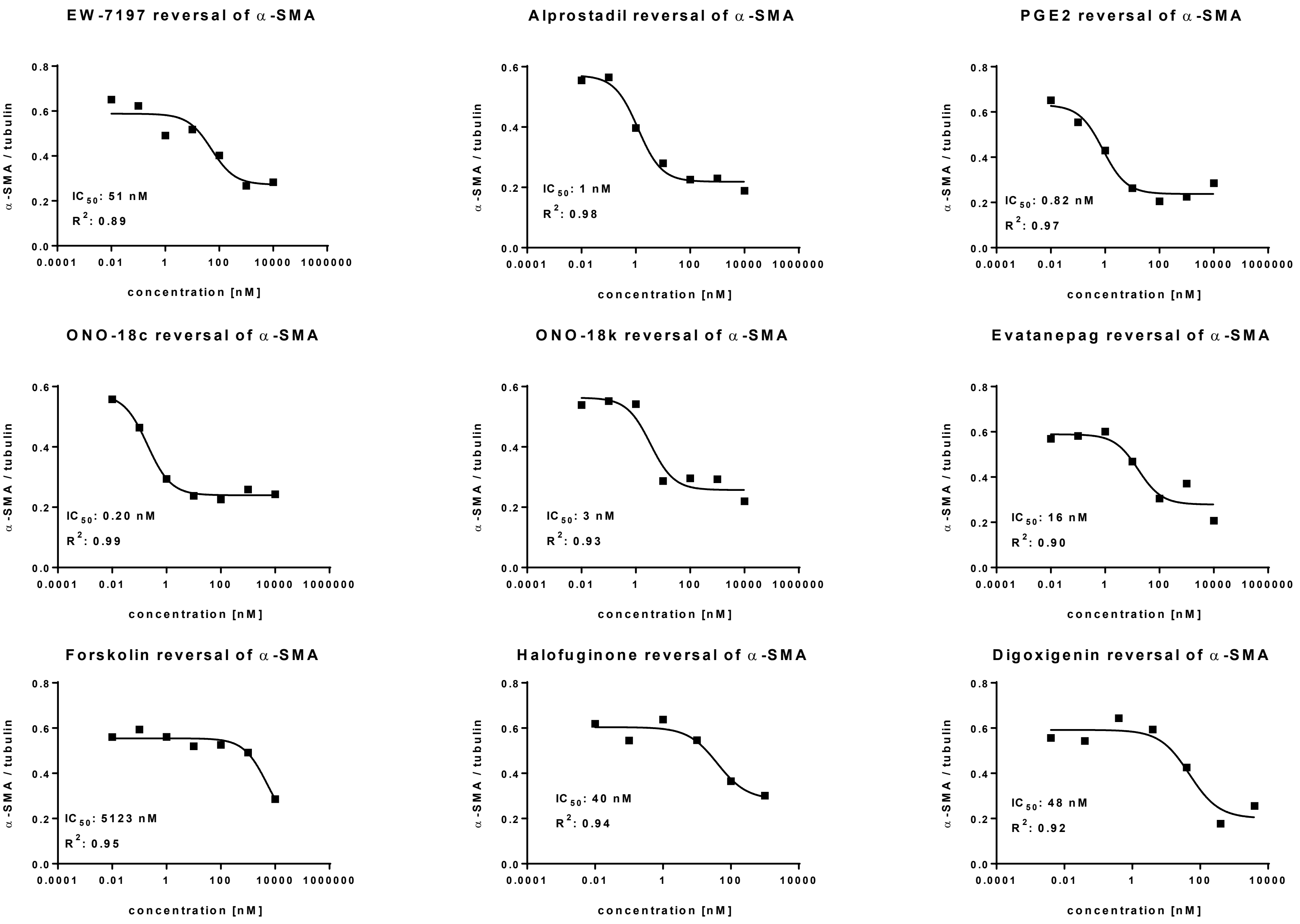
EW-7197 reversal of α-SMA
IC50: 51 nM
R2: 0.89
Alprostadil reversal of α-SMA
IC50: 1 nM
R2: 0.98
PGE2 reversal of α-SMA
IC50: 0.82 nM
R2: 0.97
ONO-18c reversal of α-SMA
IC50: 0.20 nM
R2: 0.99
ONO-18k reversal of α-SMA
IC50: 3 nM
R2: 0.93
Evatanepag reversal of α-SMA
IC50: 16 nM
R2: 0.90
Forskolin reversal of α-SMA
IC50: 5123 nM
R2: 0.95
Halofuginone reversal of α-SMA
IC50: 40 nM
R2: 0.94
Digoxigenin reversal of α-SMA
IC50: 48 nM
R2: 0.92
α-SMA / tubulin
concentration [nM]
0.0
0.2
0.4
0.6
0.8
0.0001
0.01
1
100
10000
1000000

E

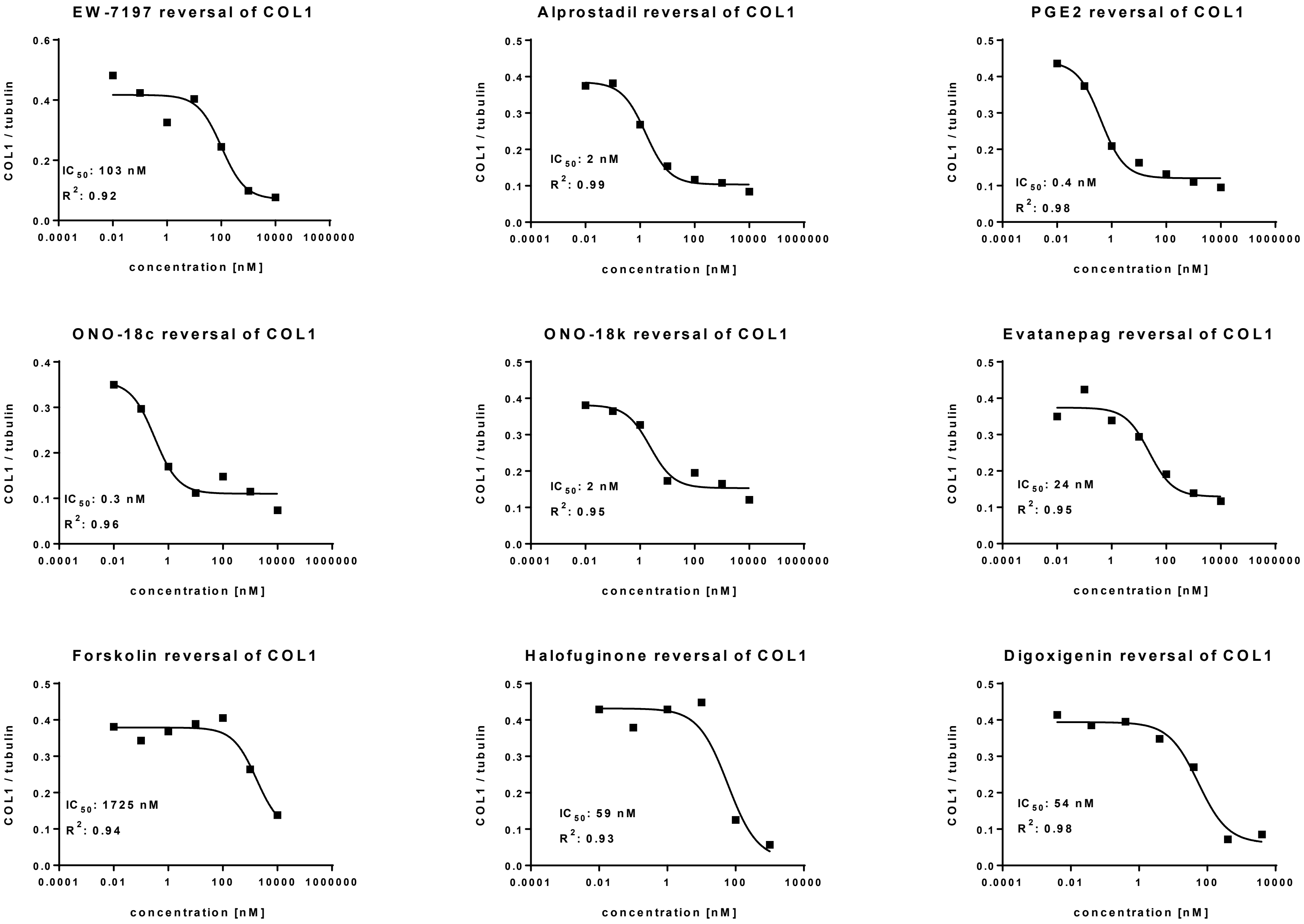
EW-7197 reversal of COL1
IC_{50}: 103 nM
R^2: 0.92
Alprostadil reversal of COL1
IC_{50}: 2 nM
R^2: 0.99
PGE2 reversal of COL1
IC_{50}: 0.4 nM
R^2: 0.98
ONO-18c reversal of COL1
IC_{50}: 0.3 nM
R^2: 0.96
ONO-18k reversal of COL1
IC_{50}: 2 nM
R^2: 0.95
Evatanepag reversal of COL1
IC_{50}: 24 nM
R^2: 0.95
Forskolin reversal of COL1
IC_{50}: 1725 nM
R^2: 0.94
Halofuginone reversal of COL1
IC_{50}: 59 nM
R^2: 0.93
Digoxigenin reversal of COL1
IC_{50}: 54 nM
R^2: 0.98
COL1 / tubulin
concentration [nM]
